# A visible-light-induced, metal-free bis-arylation of 2,5-dichlorobenzoquinone

**DOI:** 10.3762/bjoc.17.149

**Published:** 2021-09-06

**Authors:** Pieterjan Winant, Wim Dehaen

**Affiliations:** 1 Molecular Design and Synthesis, Department of Chemistry, KU Leuven, Celestijnenlaan 200F, 3001 Leuven, Belgium

**Keywords:** benzoquinone, diazonium salts, Green Chemistry, Meerwein arylation, photoredox

## Abstract

A metal-free protocol for the direct bis-arylation of 2,5-dichlorobenzoquinone with aryldiazonium salts is reported. The reactive salts are generated in situ and converted to radicals through irradiation with visible light. Reaction products precipitate from the solvent, eliminating the need for purification and thus providing a novel green method for the synthesis of versatile bis-electrophiles.

## Introduction

Quinones or quinoid-based structures are ubiquitous in nature [1–3]. These versatile structures have shown promising antimalarial [4–5], antibacterial [6], and chemotherapeutic [6–8] properties. Their inherent oxidative nature makes them crucial players in both the regulation of reactive oxygen species and biological stress levels in human tissue [9], as well as in the photosynthesis of plants and bacteria [10]. Aside from their use in therapeutics, quinones possess unique photophysical and electronic properties, resulting in a multitude of applications in dyes [11], molecular electronics [12], and oxidants or ligands in organic chemistry [13–14].

This wide range of applications has long fueled an interest in exploring innovative ways of functionalizing quinones. However, arylation of (benzo)quinones has proven to be a challenging endeavor. As conventional methods, e.g., the Heck reaction are incompatible with quinones [15], a large subset of research recently focused on radical-based CH-arylation reactions. In 2011, Baran published a seminal paper that relied on using a AgNO_3_/K_2_S_2_O_8_ induced homolytic splitting of boronic acids in a useful radical CH mono-arylation of benzoquinones [16]. Later research demonstrated that the metal catalyst can be replaced by a less expensive iron catalyst [17–18] or even be omitted at higher temperatures [19]. Despite their reputation as unstable intermediates, aryldiazonium salts have long played an essential role as radical precursors [20]. Their use in CH-arylation reactions of olefins, catalyzed by copper salts, was first published by Meerwein in 1939 [21]. Recently, we published a Meerwein arylation/cyclization sequence to benzofuropyridine derivatives in this journal [22]. Attempts at reducing aryldiazonium salts organocatalytically have also been successful [23–26]. Inspired by the seminal work by Sanford [27], König et al. [28] designed a simple and effective CH-arylation reaction combining diazonium chemistry with photoredox catalysis. By using eosin Y and green light as a reducing agent an array of substrates were functionalized.

Common among reports on the radical CH-arylation of benzoquinones is the use of multiple equivalents of starting material to prevent bis-arylation. The latter inherently relies on the use of an excess of radical precursor, potentially leading to both regioisomers and unwanted side products, complicating purification. As a result, accounts of bis-arylation using radical chemistry are scarce and report very low yields [29–32]. While transition-metal catalysis is a viable strategy, it is often based on multistep procedures requiring large amounts of toxic metal complexes [12,33–34]. Later improvements still demand prefunctionalization of the quinone substrate [35–37]. While effectively eliminating this requirement, CH bis-arylation using palladium complexes still suffers from regioselectivity issues [38–39].

Since bis-arylated quinones are promising substrates in a range of different applications (vide infra), a simple and effective method of synthesizing these types of compounds would be of great use.

Motivated by the results of König et al., we set out to develop a metal-free strategy combining photoredox and diazonium chemistry. By starting from the commercially available 2,5-dichlorobenzoquinone (**1**), we eliminate the issue of regioselectivity and effectively create a green synthesis pathway towards a versatile bis-electrophile.

## Results and Discussion

We initiated our investigation by functionalizing 2,5-dichlorobenzoquinone (**1**), using conditions similar to those reported by König et al. [28], differing only in the use of an excess of radical precursor. As expected, exposing a mixture of **1**, aryldiazonium salt **2a** and eosin Y in DMSO to green light resulted in a complex reaction mixture consisting of a mixture of mono- and bis-arylated products, accompanied by a range of unwanted side products. The complexity of the mixture resulted in the isolation of compound **3a** in a rather poor yield of 15% (Table 1, entry 1). Furthermore, this initial experiment elucidated that four equivalents (two per reaction site) of the aryldiazonium salt were required to obtain significant conversion of the starting material. Changing the solvent to acetone significantly increased the reaction yield, while dichloromethane as a solvent only resulted in trace amounts of the product (Table 1, entries 2 and 3). Due to the complex reaction mixtures, purification of the reaction product through liquid chromatography was needed, significantly encumbering the procedure. When investigating other means of purification, we observed that the bis-arylated product selectively precipitated from methanol. The latter proved to be a crucial discovery in the optimization process, since using methanol as a reaction solvent not only significantly simplified purification, but also increased the yield (Table 1, entry 4). Precipitation also occurred in acetonitrile, however, the yield was slightly lower (Table 1, entry 5).

**Table 1:** Optimization study for the bis-arylation of 2,5-dichlorobenzoquinone (**1**).^a^

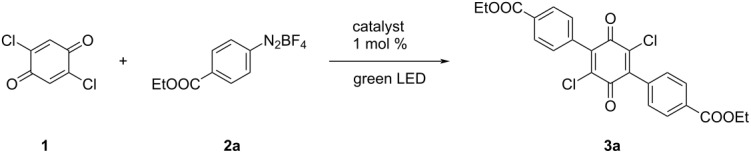				

entry	solvent	catalyst	yield (%)^b^	time (h)

1	DMSO	eosin Y	15	24
2	acetone	eosin Y	56	36
3	DCM	eosin Y	trace	24
4	methanol	eosin Y	65	12
5	acetonitrile	eosin Y	56	12
**6**	**methanol**	–	**65**	**12**
7	methanol^c^	–	55	2
8	isopropanol	–	35	24
9	ethanol	–	41	24
10	methanol^d^	–	trace	24

^a^Reaction conditions: quinone **1** (0.3 mmol), aryldiazonium salt **2a** (1.2 mmol), eosin Y (1 mol %) in solvent (2.5 mL). ^b^Isolated yield. ^c^Blue LEDs were used. ^d^Reaction in absence of irradiation source at 35 °C.

While investigating the influence of the catalyst, we discovered that the reaction proceeded smoothly in the absence of eosin Y, implying an autocatalytic or self-promoting system. A similar process has recently been described by Wu et al. [40]*,* in the arylation of BODIPYs [41–43], where radicals are formed through oxidative quenching of the excited substrate. While an excited state of **1** could react in a similar fashion, the absorption maximum of **1** is centered around 280 nm, which is outside the emission spectrum of green LEDs. A recent study by Hashmi et al. [44], however, shed a completely different light on our findings. The authors reported the arylation of different substrates by irradiating aryldiazonium salts in methanol with blue light in the absence of a catalyst. Radicals are proclaimed to be generated through excitation of an intermediate aryl cation formed in the reaction.

Eager to investigate the transferability of this concept to our system, the light source was changed to blue LEDs. This change seemed to increase reactivity, observed by significantly more vigorous nitrogen release, resulting in shortening of the reaction time to 2 h. The reaction yield on the other hand was slightly lower as compared to when green LEDs were used. Based on the report by Hashmi, we hypothesize that the overlap in wavelengths emitted by both green and blue LEDs cause the reaction to work in both instances. The diminished intensity of shorter wavelengths in the green LED causes a slower, more controlled generation of radicals resulting in a slightly higher yield compared to blue LEDs.

Aside from methanol, the reaction product also precipitates from both ethanol and isopropanol, however, no increase in yield was observed (Table 1, entries 8 and 9). A control experiment was carried out omitting the light source, while heating the reaction vessel to 35 °C, mimicking the temperature generated by the light source. This control experiment confirmed the need for a light source, as only trace amounts of reaction product were formed after 24 h without irradiation (Table 1, entry 10).

Despite the effectiveness of our strategy, the necessity of isolating the potentially hazardous diazonium salt may be seen as an important disadvantage. Arylating the quinone starting directly from the aniline in a one-pot procedure would effectively avoid the need for isolation of the potentially explosive salt and therefore greatly benefit our procedure.

The in situ formation of the aryldiazonium salt from the corresponding aniline and *tert*-butyl nitrite in methanol and subsequent exposure to green light in the presence of 2,5-dichlorobenzoquinone (**1**) proved to generate the desired product in yields that were similar compared to when using the isolated salt. A brief comparison of acids identified tetrafluoroboric acid to be the optimal proton source (65% yield), with other reagents like tosic, mesic, and hydrochloric acid affording slightly diminished (50–57%) yields (Table 2).

**Table 2:** Arylation of 2,5-dichlorobenzoquione (**1**) with anilines.^a^

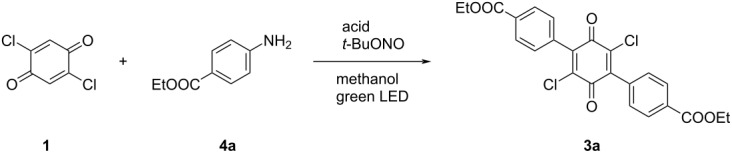		

entry	acid	yield (%)^b^

1	HBF_4_	64
2	*p*-TsOH	50
3	MsOH	55
4	HCl	57

^a^Reaction conditions: quinone **1** (0.3 mmol), aniline **4a** (1,2 mmol), acid (1.5 mmol), *tert*-butyl nitrite (*t*-BuONO, 1.5 mmol) in methanol (2.5 mL). ^b^Isolated yield.

**Scheme 1:**
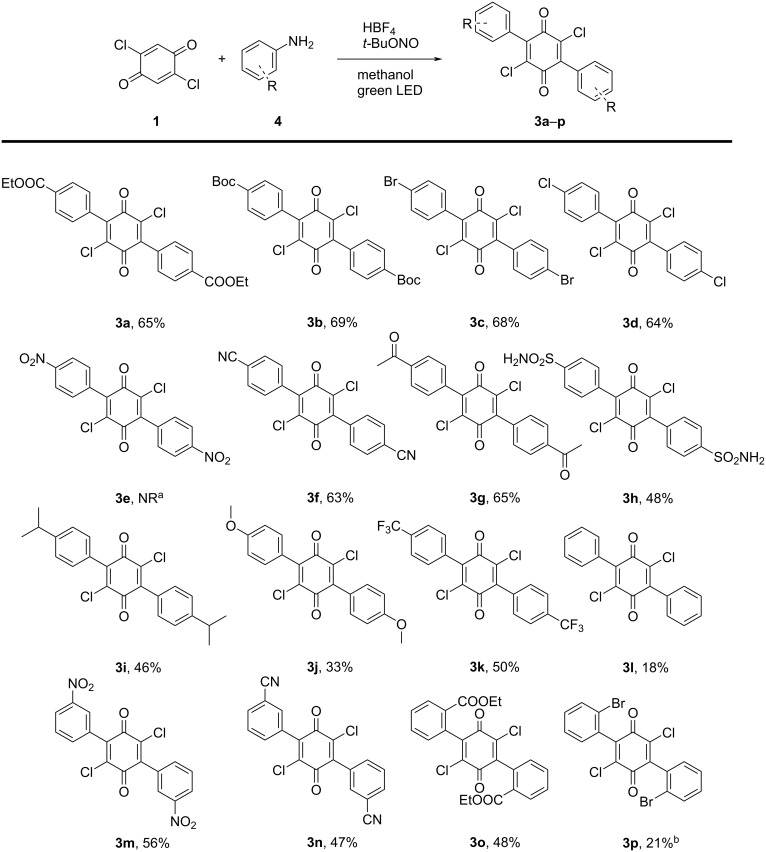
Reaction scope. Conditions: quinone **1** (0.3 mmol), aniline **4** (1.2 mmol), HBF_4_ (1.5 mmol), *t*-BuONO (1.5 mmol) in methanol (2.5 mL). ^a^No reaction. ^b^Product is unstable. Yields presented are isolated yields.

Having identified the optimal reaction conditions, different anilines were applied to explore the scope of the reaction (Scheme 1). The procedure is tolerant towards a range of different substituents. Both 4- and 3-substituted anilines readily functionalized the quinone (**3a**–**n**), and the halogens and other functional groups present are providing options for further functionalization. When 4-nitroaniline (**4e**) was used, the monoarylated product precipitated from the reaction mixture. Due to the very limited solubility of this intermediate, the latter was unable to react further. Changing the solvent to DMSO to try to tackle the solubility issue did not alter the outcome.

The diazonium salt derived from the electron-rich 4-anisidine (**4j**) proved to be less reactive, requiring 48 hours of reaction time. Compared to other substrates, reacting the chlorinated quinone with unsubstituted aniline (**4l**) resulted in substantially more side products, explaining the lowered yield. The reaction also required six equivalents and 72 h of reaction time to produce the product.

*ortho*-Substituted anilines are interesting substrates as the product enables several possibilities for post-functionalization. Despite the inherent steric hindrance originating from the *ortho*-substituent, **3o** was successfully synthesized in a yield comparable to its *para*-substituted analog **3a**. The brominated product **3p**, however, proved to be unstable.

The inability to scale-up Meerwein-type arylations is a flaw often ascribed to the explosive nature of the aryldiazonium salts. Since the strategy presented here does not involve isolating these reactive substrates, the potential risk is minimized. The procedure was safely repeated for the synthesis of **3o** on a 10 mmol (2 g) scale without altering the yield.

**Scheme 2:**
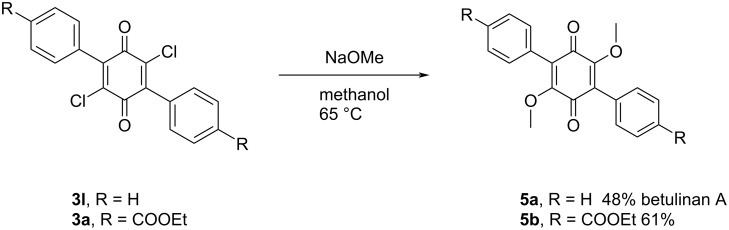
Synthesis of betulinan A and its analog **5b**. Conditions: quinone **3** (0.1 mmol), NaOMe (0.25 mmol) in methanol (1 mL).

We further demonstrated the synthetic utility of the method by successfully synthesizing betulinan A, a natural product isolated from *Lenzites betulina* [2], in two steps starting from commercially available starting materials. Analogs of the natural product **5a**, such as **5b** can also readily be prepared from the corresponding diarylquinone **3a** (Scheme 2).

## Conclusion

In summary, we have developed a straightforward metal-free procedure for the synthesis of bis-arylated 2,5-dichlorobenzoquinones. The reaction is tolerant towards a wide range of functional groups, occurs under mild conditions in an environmentally benign solvent and does not require further purification of the reaction product. The reactive aryldiazonium salt is synthesized in situ, minimizing risks and enabling scale-up. The chlorine substituents on the quinone were successfully functionalized as part of the synthesis of natural product betulinan A, proving that this work may further facilitate the synthesis of new products or products that would otherwise be difficult to prepare.

## Supporting Information

File 1 Experimental part and NMR spectra.
