# Supplementary material for: A visible-light-induced, metal-free bis-arylation of 2,5-dichlorobenzoquinone
Source: Beilstein J Org Chem. 2021 Sep 6;17:2315–20. doi: 10.3762/bjoc.17.149 (PMC8450952; doi:10.3762/bjoc.17.149)
Supplement: File 1 — Experimental part and NMR spectra. [file Beilstein_J_Org_Chem-17-2315-s001.pdf]

BEILSTEIN JOURNAL OF ORGANIC CHEMISTRY

Supporting Information

for

A visible-light-induced, metal-free bis-arylation of 2,5-dichlorobenzoquinone

Pieterjan Winant and Wim Dehaen

Beilstein J. Org. Chem. **2021,** *17,* 2315–2320. doi:10.3762/bjoc.17.149

Experimental part and NMR spectra

License and Terms: This is a supporting information file under the terms of the Creative Commons Attribution License (https://creativecommons.org/licenses/by/4.0). Please note that the reuse, redistribution and reproduction in particular requires that the author(s) and source are credited and that individual graphics may be subject to special legal provisions.
The license is subject to the *Beilstein Journal of Organic Chemistry* terms and conditions: (https://www.beilstein-journals.org/bjoc/terms)

1. General

Chemicals received from commercial sources (Sigma-Aldrich, Acros Organics, J&K Scientific, Alfa Aesar or TCI Chemicals) were used without further purification. All reactions were carried out under argon atmosphere. Dry reaction solvents were purchased from commercial sources. Thin-layer chromatography (TLC) was performed on silica gel 0.20 mm 60 with fluorescent indicator UV254 (pre-coated aluminum sheets) from Merck. For column chromatography 60–200 mesh silica gel 60 (Acros) was used as stationary phase. NMR spectra were acquired on commercial instruments (Bruker Avance 300 MHz, Bruker AMX 400 MHz, or Bruker Avance II+ 600 MHz) and chemical shifts (δ) are reported in parts per million (ppm) referenced to tetramethylsilane (^{1}H), or the internal (NMR) solvent signal (^{13}C). High resolution mass spectra were acquired on a quadrupole orthogonal acceleration time-of-flight mass spectrometer equipped with an APCI source (Synapt G2 HDMS, Waters, Milford, MA, USA). Samples were infused at 200 µL/min and spectra were obtained in positive mode with a resolution of 15000 (FWHM) using leucine enkephalin as lock mass.

2. Experimental methods

General procedure for the arylation of 2,5-dichlorobenzoquinone

To a screw-capped reaction tube equipped with a magnetic stirring bar, aniline (**4**, 1.12 mmol) was added in 2 mL of methanol. The solution was cooled to 0 °C and a 48 wt % solution of tetrafluoroboric acid (0.17 mL, 1.4 mmol) and *tert*-butyl nitrite (0,17 mL, 1.4 mmol) were added. The reaction mixture was allowed to stir for 15 minutes after which 2,5-dichlorobenzoquinone (**1**, 50mg, 0.28 mmol) was added. If necessary, 0.5 mL of methanol were added to allow for mixing of the quinone with the diazonium salt. The reaction tube was purged with argon and irradiated overnight under an argon atmosphere

with a green LED. The reaction vessel was cooled using pressurized air to prevent significant heating of the reaction medium. Upon completion of the reaction (as checked by TLC analysis), the mixture was filtered and the precipitate dried under vacuum. Where necessary, the filtered product was recrystallized from methanol.

Diethyl 3',6'-dichloro-2',5'-dioxo-2',5'-dihydro-[1,1':4',1''-terphenyl]-4,4''-dicarboxylate (3a)

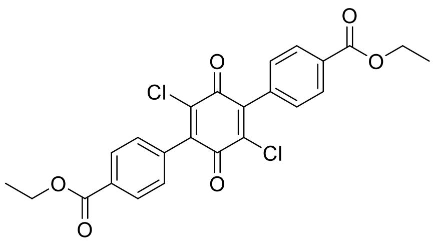

Yield: 65% (86 mg)

Yellow solid – MP: 246-249°C. Reaction time: 12h. ^{1}H NMR (400 MHz, $CDCl_3$) δ (ppm) 8.18 (d, *J* = 8.2 Hz, 4H), 7.44 (d, *J* = 8.2 Hz, 4H), 4.42 (q, *J* = 7,1 Hz, 4H), 1.42 (t, *J* = 7,1 Hz, 6H). ^{13}C NMR (101 MHz, $CDCl_3$): δ (ppm) 176.80, 165.83, 142.87, 141.37, 134.89, 131.77, 129.72, 129.46, 61.34, 14.33

Spectra in accordance with available literature data[1].

Di-*tert*-butyl 3',6'-dichloro-2',5'-dioxo-2',5'-dihydro-[1,1':4',1''-terphenyl]-4,4''-dicarboxylate (3b)

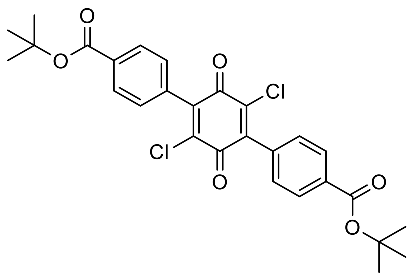

Yield: 69% (89 mg)

Yellow solid – MP: 300+ °C. Reaction time: 12h. ^{1}H NMR (400 MHz, $CDCl_3$): δ (ppm) 8.12 (d, *J* = 8.3 Hz, 4H), 7.41 (d, *J* = 8.3 Hz, 4H), 1.62 (s, 18H). ^{13}C NMR (101 MHz, $CDCl_3$): δ (ppm) 176.84, 164.92, 142.97, 141.29, 134.52, 133.28, 129.57, 129.33, 81.85, 28.18 HRMS (APCI-Q-TOF): calculated for $C_{28}H_{26}Cl_2O_6$ [M-H]-: 527.1034 found 527.1008.

4,4''-Dibromo-3',6'-dichloro-[1,1':4',1''-terphenyl]-2',5'-dione (3c)

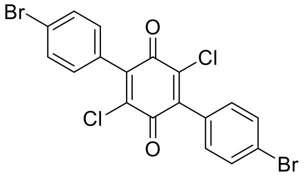

Yield: 68% (94 mg)

Yellow solid – MP: 300+ °C. Reaction time: 12h. ^{1}H NMR (400 MHz, DMSO): δ (ppm) 7.75 (d, *J* = 8.50 Hz, 4H), 7.34 (d, *J* = 8.50 Hz, 4H). ^{13}C NMR (101 MHz, DMSO): δ (ppm) 177.14, 142.49, 140.21, 132.12, 131.72, 131.24, 123.46. HRMS (APCI-Q-TOF): calculated for $C_{18}H_8Br_2Cl_2O_2$ [M-H]-: 482.8196 found 482.8151

3',4,4'',6'-Tetrachloro-[1,1':4',1''-terphenyl]-2',5'-dione (3d)

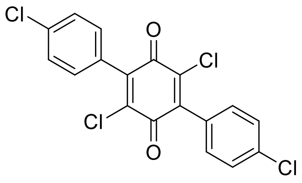

Yield: 64% (72 mg)

Yellow solid – MP: 293-295°C. Reaction time: 12h. ^{1}H NMR (400 MHz, DMSO): δ (ppm) 7.61 (d, *J* = 8.42 Hz, 4H), 7.41 (d, *J* = 8.42 Hz, 4H). ^{13}C NMR (101 MHz, DMSO): δ (ppm) 177.21, 142.46, 140.30, 134.72, 131.92, 130.86, 128.80. HRMS (APCI-Q-TOF): calculated for $C_{18}H_{8}Cl_{4}O_{2}$ [M-H]-: 394.9206 found 394.9226.

3',6'-Dichloro-2',5'-dioxo-2',5'-dihydro-[1,1':4',1''-terphenyl]-4,4''-dicarbonitrile (3f)

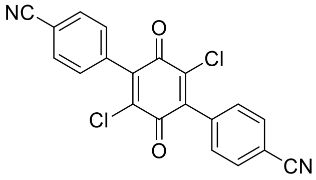

Yield: 63% (68 mg)

Yellow solid – MP: 300°C. Reaction time: 12h. ^{1}H NMR (400 MHz, DMSO): δ (ppm) 8.03 (d, *J* = 7.56 Hz, 4H), 7.59 (d, *J* = 7.56 Hz, 4H). ^{13}C NMR (101 MHz, DMSO): δ (ppm) 176.75, 142.21, 140.62, 136.75, 132.71, 130.97, 118.89, 112.66. HRMS (APCI-Q-TOF): calculated for $C_{20}H_{8}Cl_{2}N_{2}O_{2}$ [M]-: 377.9963 found 377.9952.

4,4''-Diacetyl-3',6'-dichloro-[1,1':4',1''-terphenyl]-2',5'-dione (3g)

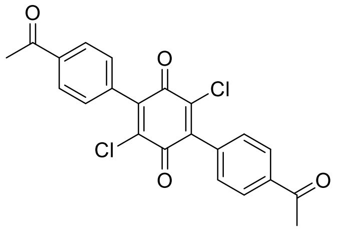

Yield: 65% (76 mg)

Yellow solid – MP: 285-287°C. Reaction time: 12h. ^{1}H NMR (400 MHz, DMSO): δ (ppm) 8.11 (d, *J* = 8.47 Hz, 4H), 7.50 (d, *J* = 8.45 Hz, 4H), 2.69 (s, 6H). ^{13}C NMR (101 MHz, DMSO): δ (ppm) 197.30, 176.75, 142.76, 141.41, 137.90, 135.10, 130.02, 128.19, 26.74. HRMS (APCI-Q-TOF): calculated for $C_{22}H_{14}Cl_2O_4$ [M]-: 412.0269 found 412.0263.

3',6'-Dichloro-2',5'-dioxo-2',5'-dihydro-[1,1':4',1''-terphenyl]-4,4''-disulfonamide (3h)

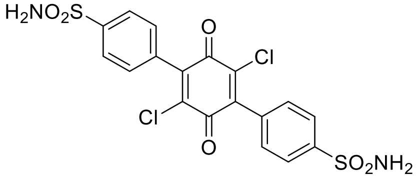

Yield: 48% (66 mg)

Yellow solid – MP: 300+°C. Reaction time: 12h ^{1}H NMR (400 MHz, DMSO): δ (ppm) 7.98 (d, *J* = 8.3 Hz, 2H), 7.58 (d, *J* = 8.3, 2H), 7.51 (s, 4H). ^{13}C NMR (101 MHz, DMSO): δ (ppm) 177.06, 145.17, 142.52, 140.54, 135.37, 130.61, 126.06. HRMS (ESI-Q-TOF): calculated for $C_{18}H_{12}Cl_2N_2O_6S_2$ [M]-: 485.9514 found 485.9509.

3',6'-Dichloro-4,4''-diisopropyl-[1,1':4',1''-terphenyl]-2',5'-dione (3i)

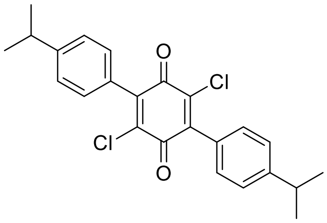

Yield: 46% (53 mg)

Yellow solid – MP: 276-277°C. Reaction time: 12h. ^{1}H NMR (400 MHz, $CDCl_3$): δ (ppm) 7.38 (d, *J* = 8.25 Hz, 4H), 7.33 (d, *J* = 8.41 Hz, 4H), 1.34 (s, 6H), 1.32 (s, 6H). ^{13}C NMR (101 MHz, $CDCl_3$): δ (ppm) 177.74, 150.83, 143.36, 140.41, 129.85, 128.18, 126.29, 34.09, 23.78. HRMS (APCI-Q-TOF): calculated for $C_{24}H_{22}Cl_2O_2$ [M-H]-: 411.0924 found 411.0909.

3',6'-Dichloro-4,4''-dimethoxy-[1,1':4',1''-terphenyl]-2',5'-dione (3j)

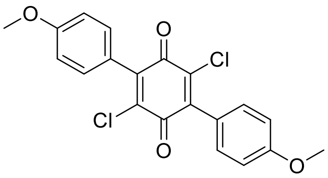

Yield: 33% (36 mg)

Yellow solid – MP: 250-252°C. Reaction time: 48h. ^{1}H NMR (400 MHz, $CDCl_3$): δ (ppm) 7.34 (d, *J* = 8.8 Hz, 4H), 7.01 (d, *J* = 8.8 Hz, 4H), 3.88 (s, 6H). ^{13}C NMR (101 MHz, $CDCl_3$): δ (ppm) 177.86, 160.81, 142.94, 139.83, 131.70, 122.94, 113.66, 55.37

Spectra in accordance with available literature data[2]

3',6'-Dichloro-4,4''-bis(trifluoromethyl)-[1,1':4',1''-terphenyl]-2',5'-dione (3k)

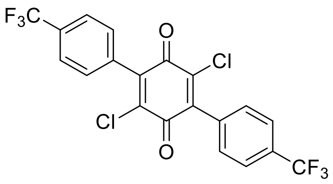

Yield: 50% (66 mg)

Yellow solid – MP: 238-240°C. Reaction time: 12h. ^{1}H NMR (400 MHz, DMSO): δ (ppm) 7.93 (d, *J* = 8.14 Hz, 4H), 7.62 (d, *J* = 8.00 Hz, 4H). ^{13}C NMR (101 MHz, DMSO) δ 176.98, 142.38, 140.64, 136.27-136.23 (m), 130.09 (q, *J* = 32.0 Hz), 125.77-125.58 (m), 124.48 (q, *J* = 272.4 Hz). HRMS (APCI-Q-TOF): calculated for $C_{20}H_8Cl_2F_6O_2$ [M]-: 463.9805 found 463.9801.

3',6'-Dichloro-[1,1':4',1''-terphenyl]-2',5'-dione (3l)

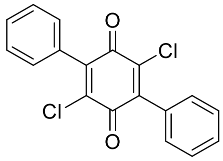

Compound **3l** was prepared using a modified version of the general procedure:

To a screw-capped reaction tube equipped with a magnetic stirring bar, aniline (153 mg, 1.68 mmol) was added in 2 mL of methanol. The solution was cooled to 0 °C and a 48 wt % solution of tetrafluoroboric acid (0.26 mL, 2.1 mmol) and *tert*-butyl nitrite (0.26 mL, 2.1 mmol) were added. The reaction mixture was allowed to stir for 15 minutes after which 2,5-dichlorobenzoquinone (**1**, 50 mg, 0.28 mmol) was added. The reaction tube was purged with argon and irradiated under an argon atmosphere with a green LED for 72 h.

Upon completion of the reaction (as checked by TLC analysis), the mixture was filtered and the precipitate dried under vacuum.

Yield 18% (17 mg)

Yellow solid – MP: 209-211°C. ^{1}H NMR (400 MHz, $CDCl_3$): δ (ppm) 7.52-7.49 (m, 6H), 7.37-7.35 (m, 4H). ^{13}C NMR (101 MHz, DMSO): δ (ppm) 177.48, 143.46, 140.87, 130.83, 129.88, 129.68, 128.25.

Spectra in accordance with available literature data[2]

3',6'-Dichloro-3,3''-dinitro-[1,1':4',1''-terphenyl]-2',5'-dione (3m)

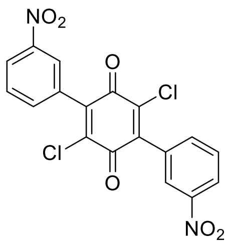

Yield: 56% (66 mg)

Yellow solid – MP: 276-278°C. Reaction time: 12h. ^{1}H NMR (400 MHz, DMSO): δ (ppm) 8.41-8.39 (m, 2H), 8.28 (s, 2H), 7.91-7.87 (m, 4H). ^{13}C NMR (101 MHz, DMSO): δ (ppm) 176.34, 147.51, 141.10, 140.44, 136.13, 132.90, 130.16, 124.38. HRMS (APCI-Q-TOF): calculated for $C_{18}H_8Cl_2N_2O_6$ [M]-: 417.9759 found 417.9755.

3',6'-Dichloro-2',5'-dioxo-2',5'-dihydro-[1,1':4',1''-terphenyl]-3,3''-dicarbonitrile (3n)

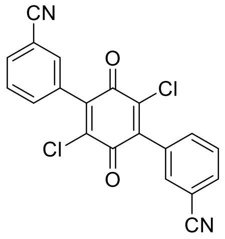

Yield: 47% (50 mg)

Yellow solid – MP: 300+°C. Reaction time: 12h. ^{1}H NMR (400 MHz, DMSO): δ (ppm) 8.01 (dt, *J* = 1.6 Hz, 2H), 7.86 (m, 2H), 7.79 (m, 2H), 7.75 (dt, *J* = 7.9, 1.6 Hz, 2H). ^{13}C NMR (101 MHz, DMSO): δ (ppm) 176.82, 141.73, 140.75, 134.87, 133.71, 133.46, 133.12, 130.12, 118.69, 111.93. HRMS (APCI-Q-TOF): calculated for $C_{20}H_8Cl_2N_2O_2$ [M]-: 377.9963 found 377.9957.

Diethyl 3',6'-dichloro-2',5'-dioxo-2',5'-dihydro-[1,1':4',1''-terphenyl]-2,2''-dicarboxylate (3o)

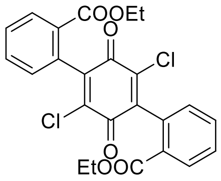

Yield: 48% (65 mg)

Yellow solid – MP: 209-211°C. Reaction time: 12h. ^{1}H NMR (400 MHz, $CDCl_3$): δ (ppm). 8.24 (ddd, *J,* 9.3, 7.9, 1.0, 2H), 7.71 (tdd, *J* = 7.6, 4.4, 1.4 Hz, 2H), 7.61 (tdd, *J* = 7.7, 2.4, 1.4 Hz, 2H), 7.45 (dd, J = 7.6, 0.9 Hz, 1H), 7.36 (dd, J = 7.6, 1.0 Hz, 1H), 4.34 (m, 4H), 1.36 (m, 6H). ^{13}C NMR (101 MHz, $CDCl_3$): δ (ppm) 177.21, 176.92, 156.69, 165.56, 146.23, 146.18, 138.35, 138.08, 133,43, 133.08, 132.63, 132.55, 130.97, 130.73, 130.23, 129.99, 129.74, 129.67, 129.33, 61.62, 61.47, 14.16. HRMS (APCI-Q-TOF): no ionization occurred.

General procedure for the synthesis of betulinan A (5a) and analog 5b

To an oven-dried, screw-capped reaction tube equipped with a magnetic stirring bar, quinone (**3**, 0.1 mmol) and NaOMe (0.25 mmol) was added in 1 mL of dry methanol. The

reaction tube was purged with argon, heated to reflux and allowed to stir for 1 h. Upon completion of the reaction (as checked by TLC analysis), the mixture was filtered and the precipitate dried under vacuum.

3',6'-Dimethoxy-[1,1':4',1''-terphenyl]-2',5'-dione (5a)

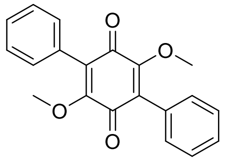

Yield: 48% (15 mg)

Yellow solid – MP: 148-150°C. Reaction time: 1h. ^{1}H NMR (400 MHz, $CDCl_3$): δ (ppm). 7.47 – 7.32 (m, 10H), 3.83 (s, 6H). ^{13}C NMR (101 MHz, $CDCl_3$): δ (ppm) 183.48, 154.70, 130.56, 130.17, 128.62, 127.96, 126.60, 61.61.

Spectra in accordance with available literature data [3].

Diethyl 3',6'-dimethoxy-2',5'-dioxo-2',5'-dihydro-[1,1':4',1''-terphenyl]-4,4''-dicarboxylate (5b)

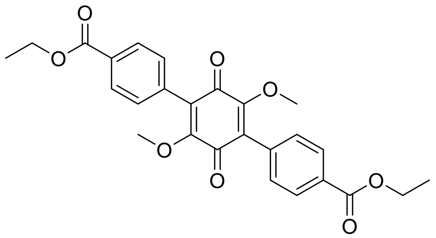

Yield: 61% (32 mg)

Yellow solid – MP: 174-176°C. Reaction time: 1h. ^{1}H NMR (400 MHz, $CDCl_3$): δ (ppm). 8.12 (d, *J* = 8.4 Hz, 4H), 7.42 (d, *J* =8.4 Hz), 4.41 (q, *J* = 7.1 Hz, 4H), 3.88 (s, 6H), 1.41 (t, *J* = 7.1 Hz, 6H). ^{13}C NMR (101 MHz, $CDCl_3$): δ (ppm) 182.70, 166.17, 154.87, 134.72,

130.63, 130.55, 129.10, 125.73, 61.82, 61.13, 14.36. HRMS (APCI-Q-TOF): calculated for $C_{26}H_{24}O_8$ [M+H]+: 465.1543 found 465.1526.

3a (^{1}H NMR, 400 MHz, $CDCl_3$)

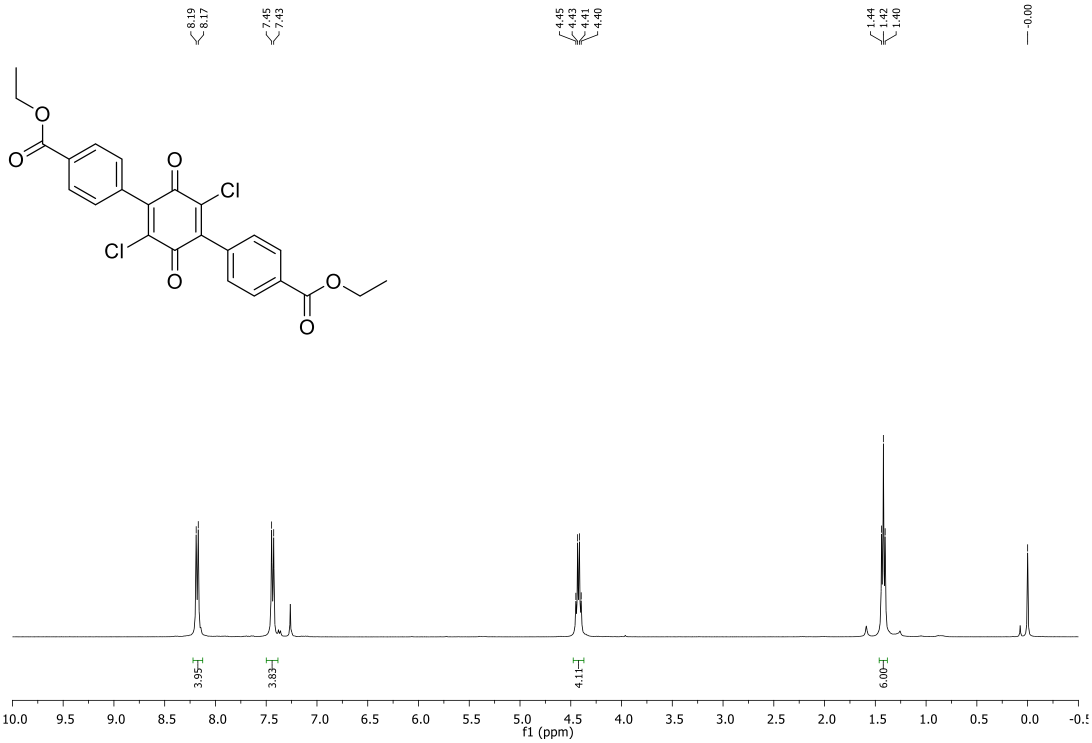

3a (^{13}C NMR, 101 MHz, $CDCl_3$)

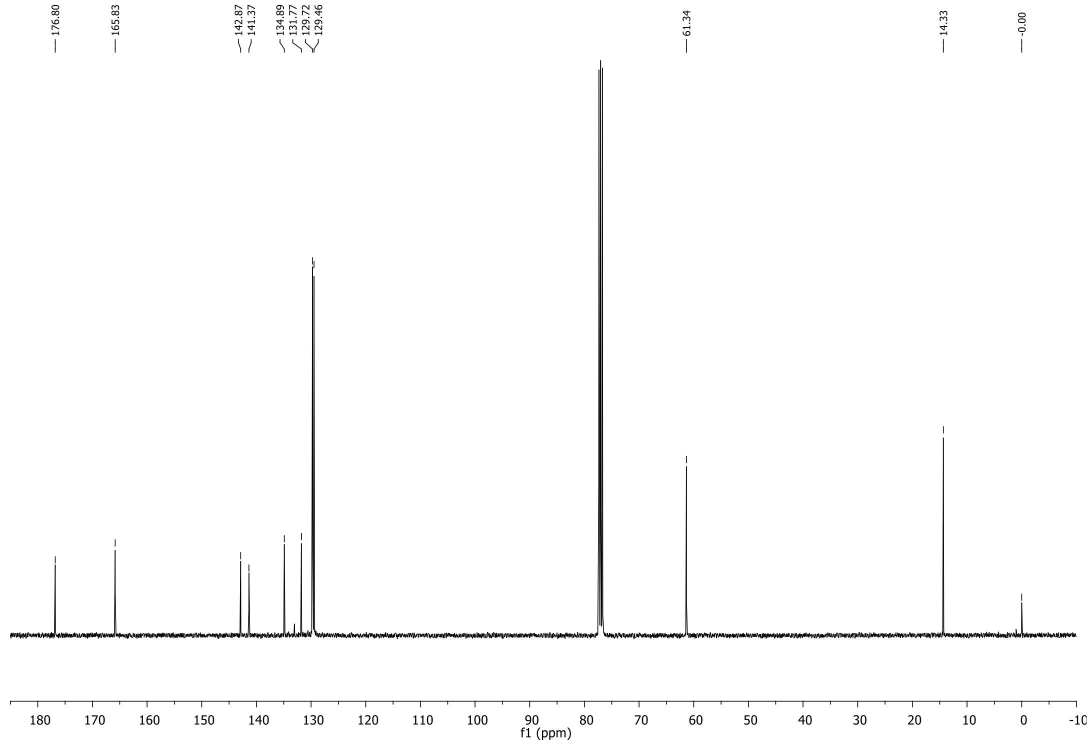

3b (^{1}H NMR, 400 MHz, $CDCl_3$)

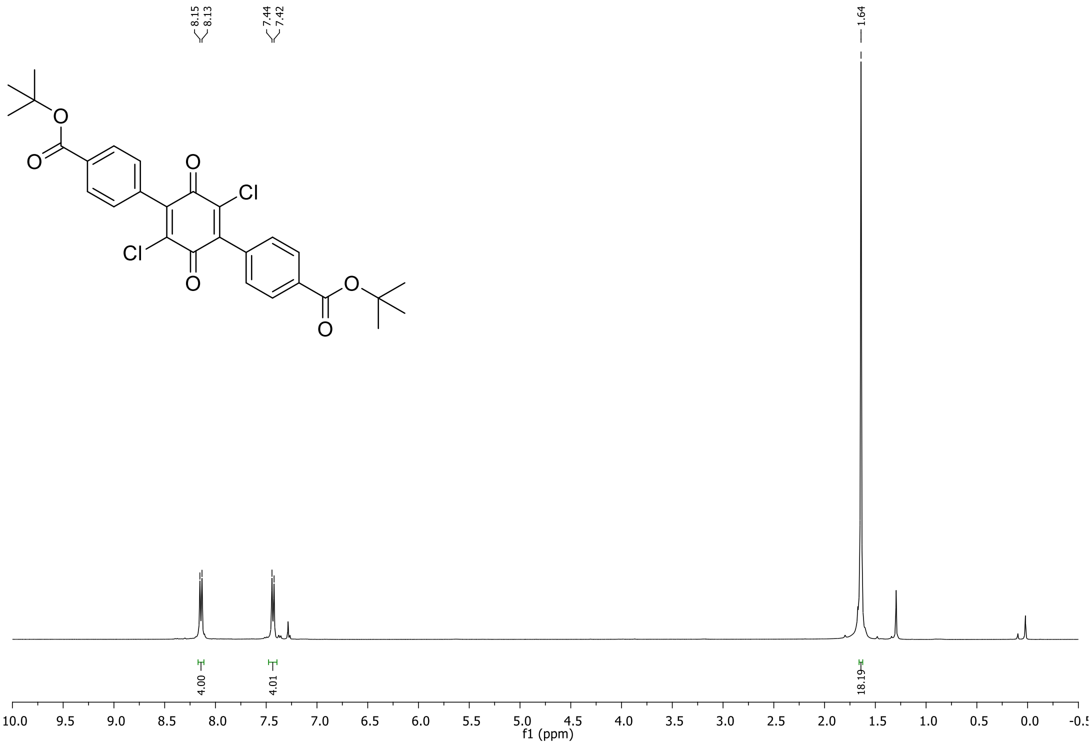

3b (^{13}C NMR, 101 MHz, $CDCl_3$)

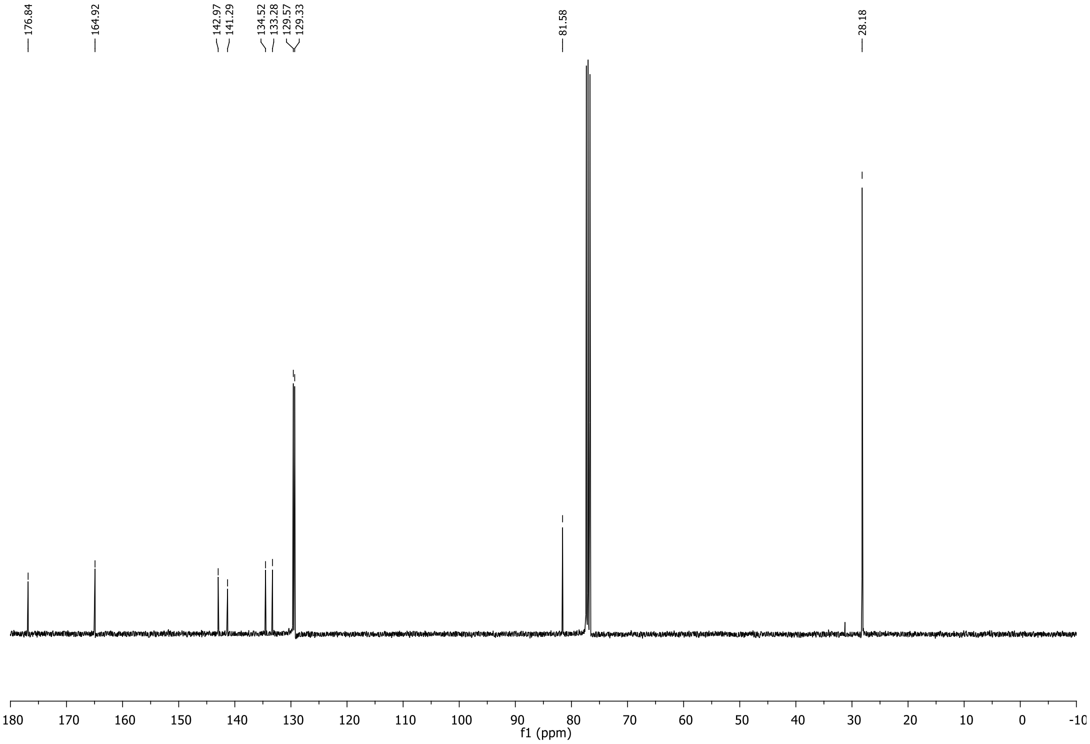

3c (^{1}H NMR, 400 MHz, DMSO)

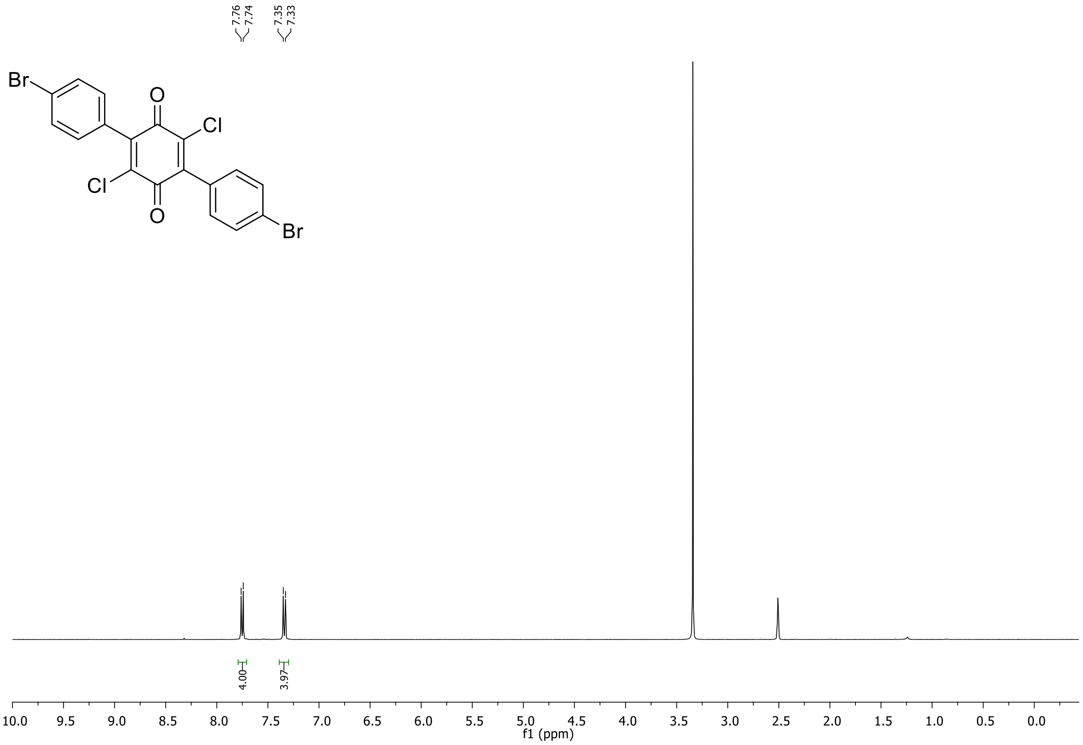

3c (^{13}C NMR, 101 MHz, DMSO)

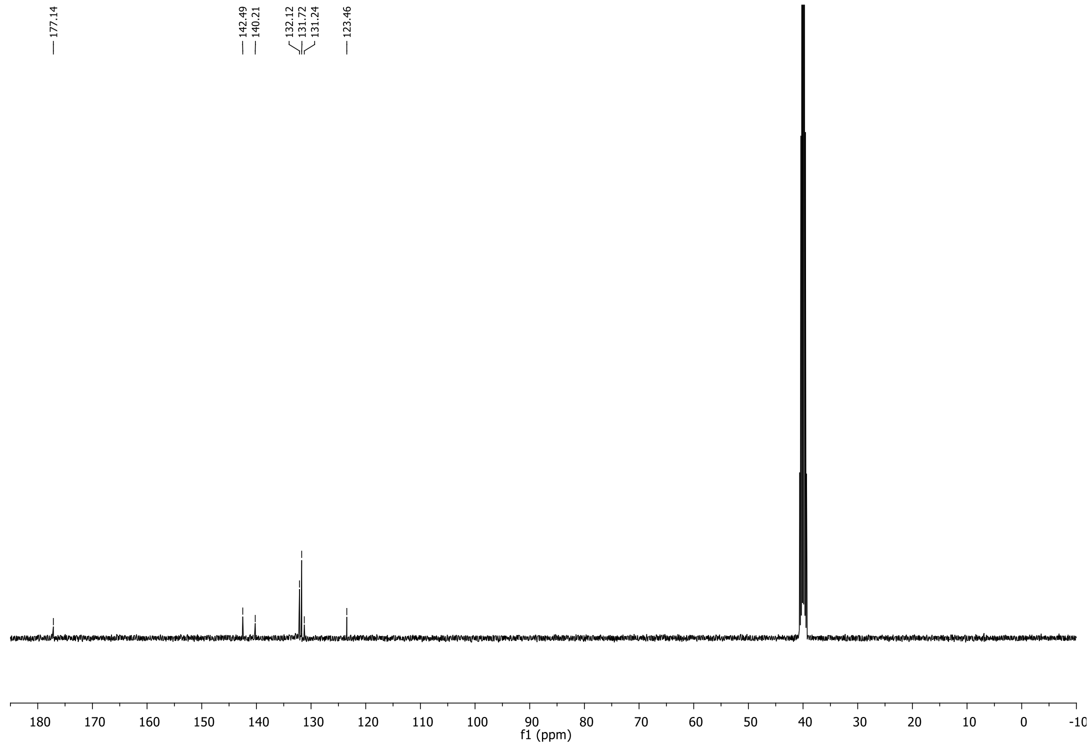

3d (^{1}H NMR, 400 MHz, DMSO)

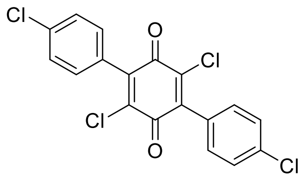

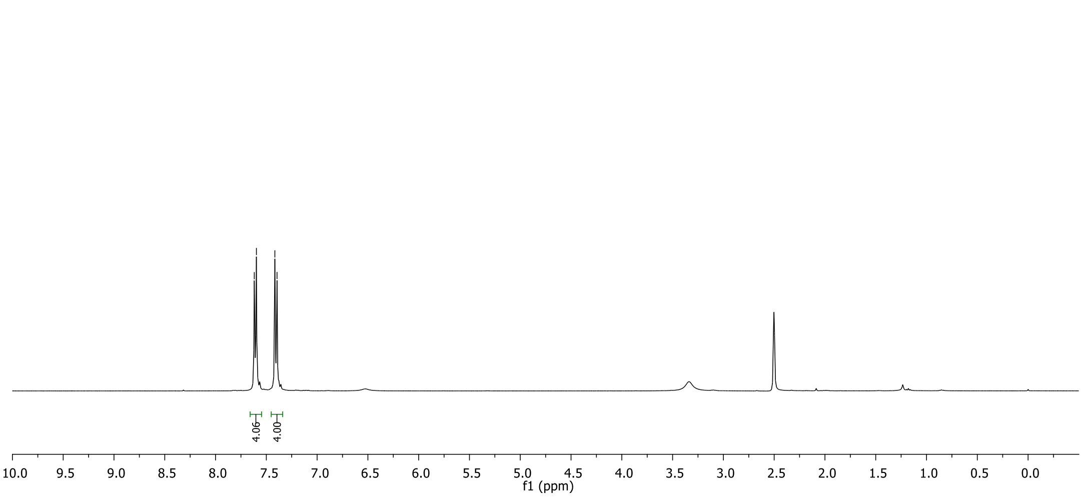

3d (^{13}C NMR, 101 MHz, DMSO)

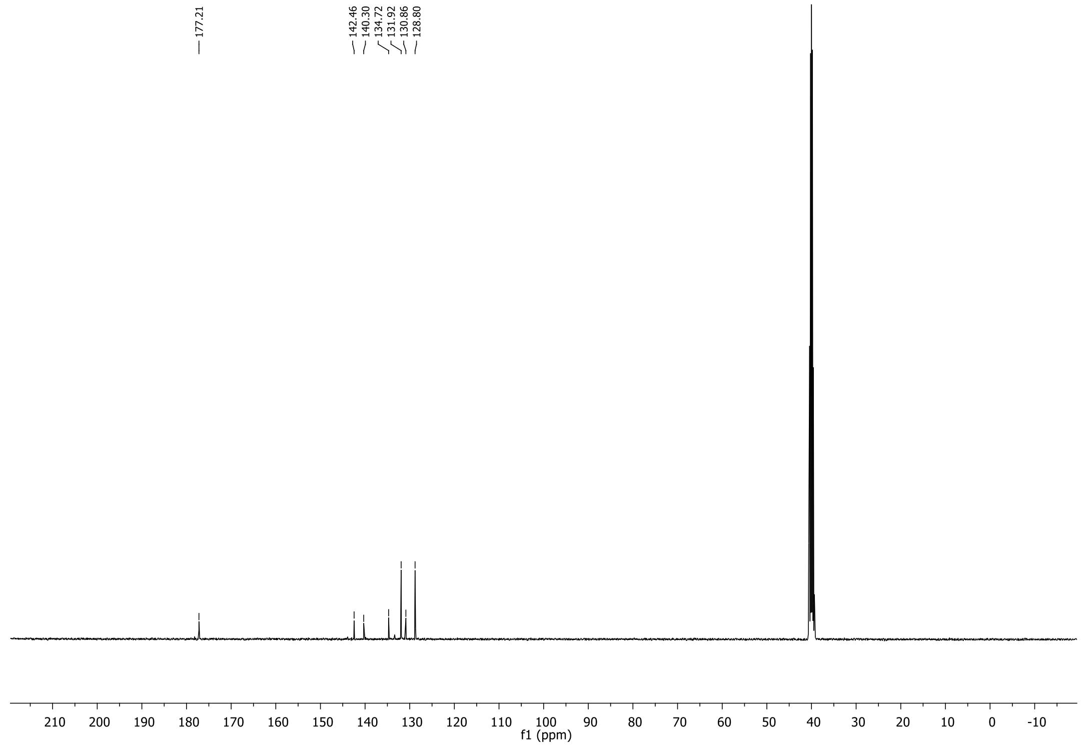

3f (^{1}H NMR, 400 MHz, DMSO)

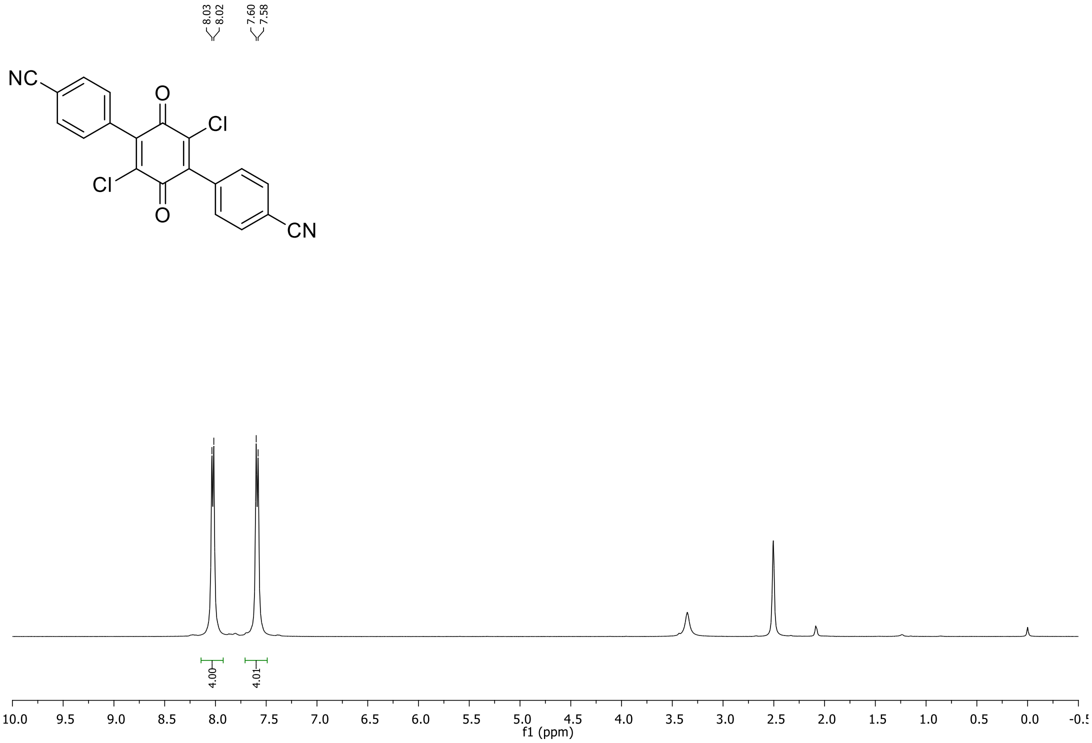

3f (^{13}C NMR, 101 MHz, DMSO)

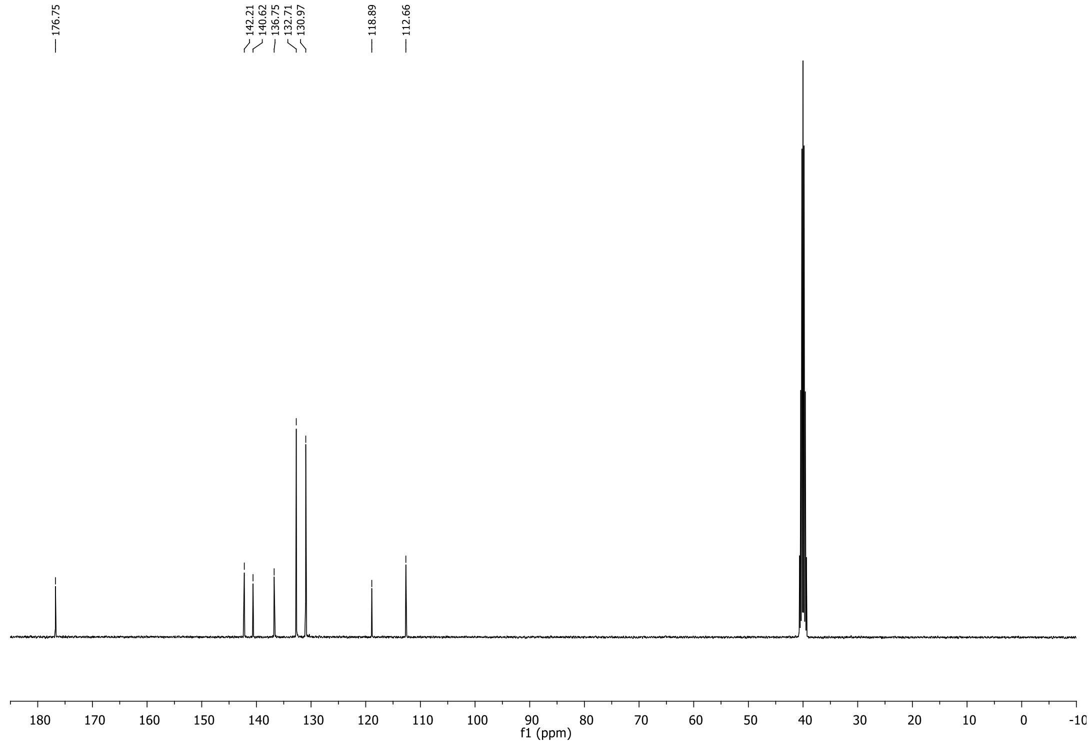

3g (^{1}H NMR, 400 MHz, CDCl$_3$)

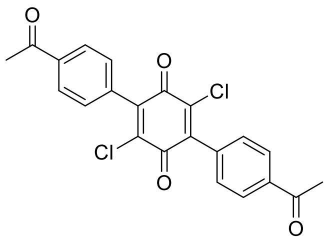

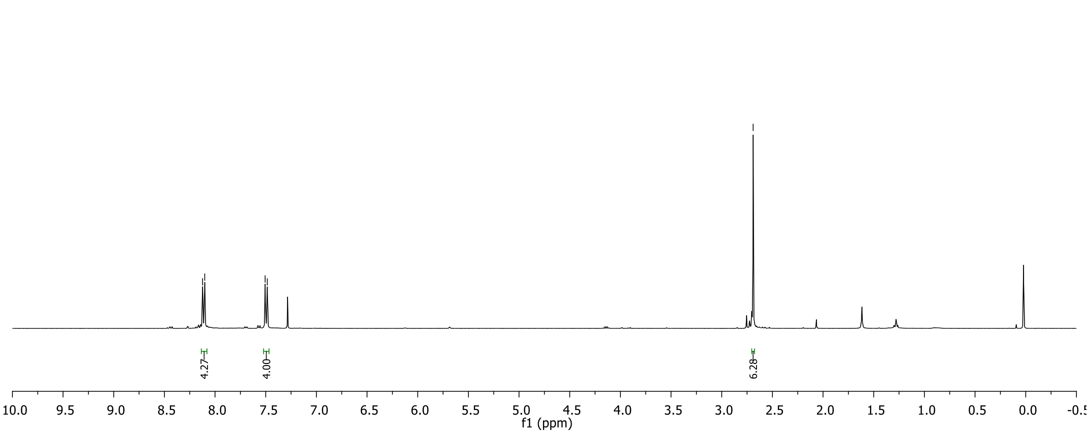

3g (^{13}C NMR, 101 MHz, CDCl$_3$)

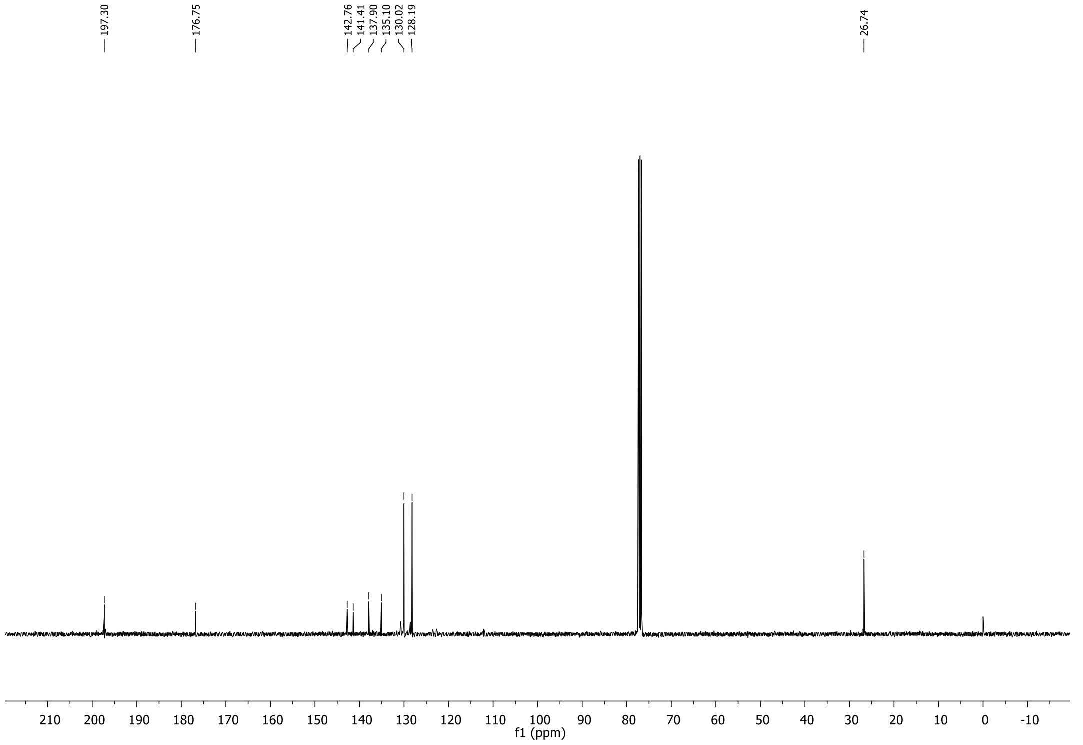

3h (^{1}H NMR, 400 MHz, $CDCl_3$)

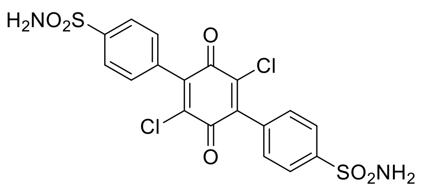

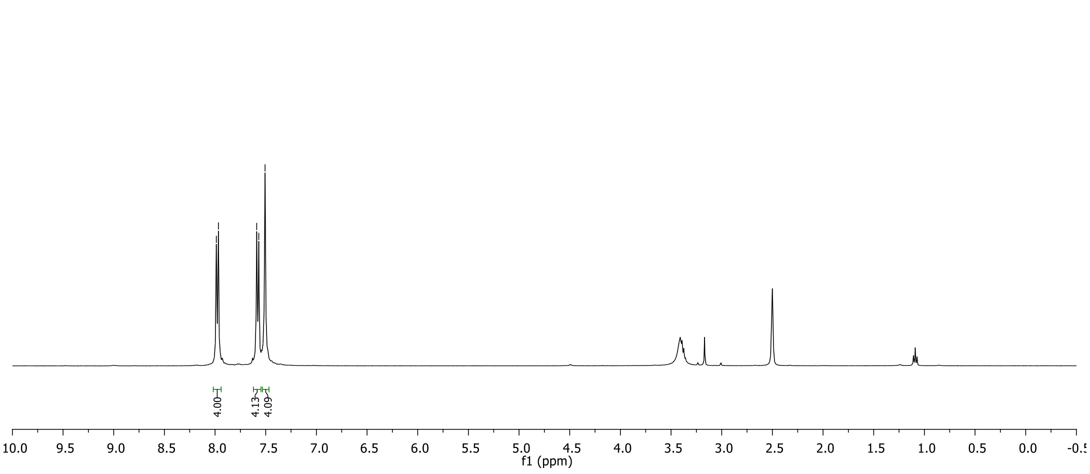

3h (^{13}C NMR, 101 MHz, $CDCl_3$)

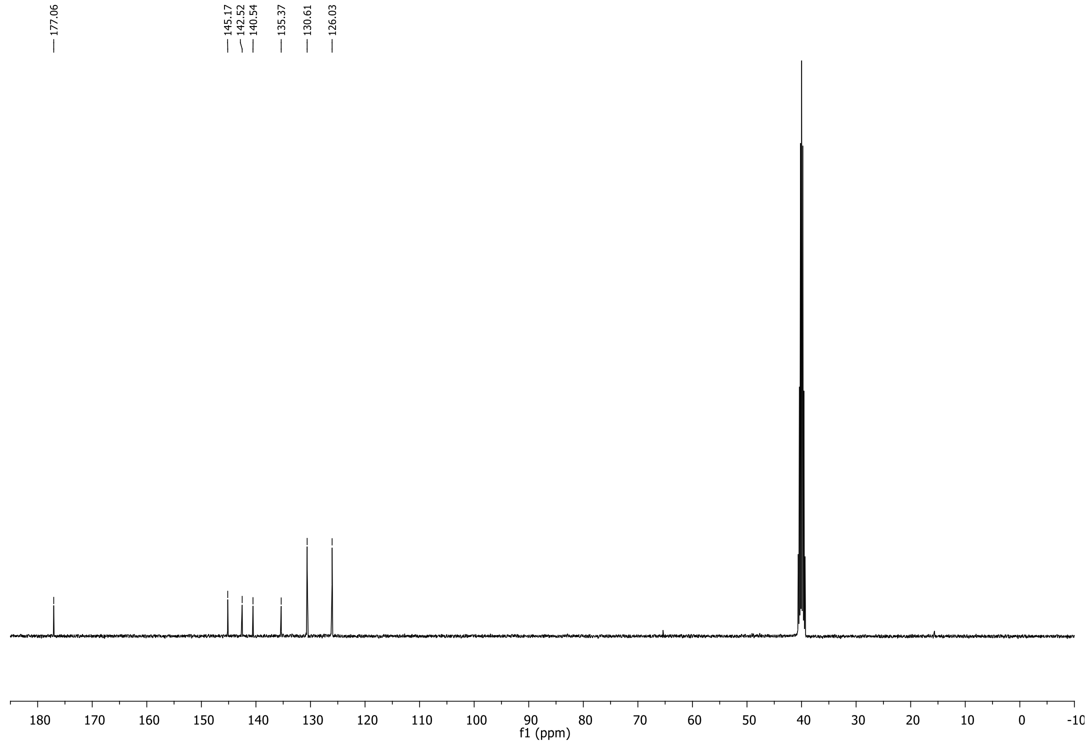

3i (^{1}H NMR, 400 MHz, $CDCl_3$)

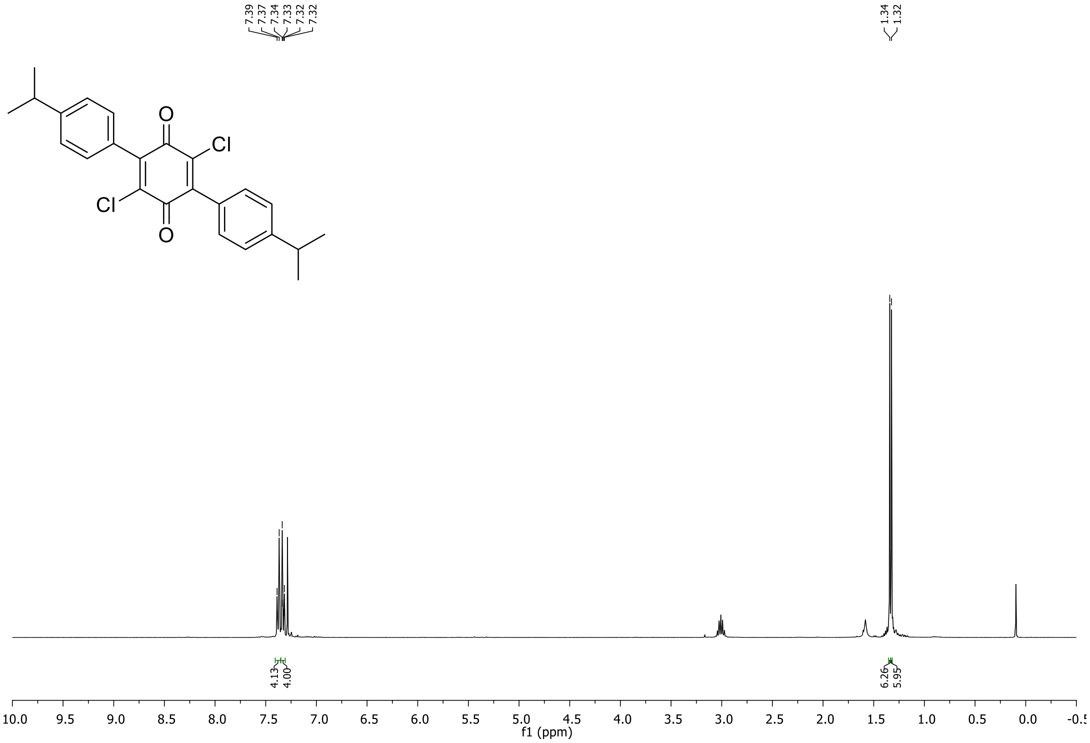

3i (^{13}C NMR, 101 MHz, $CDCl_3$)

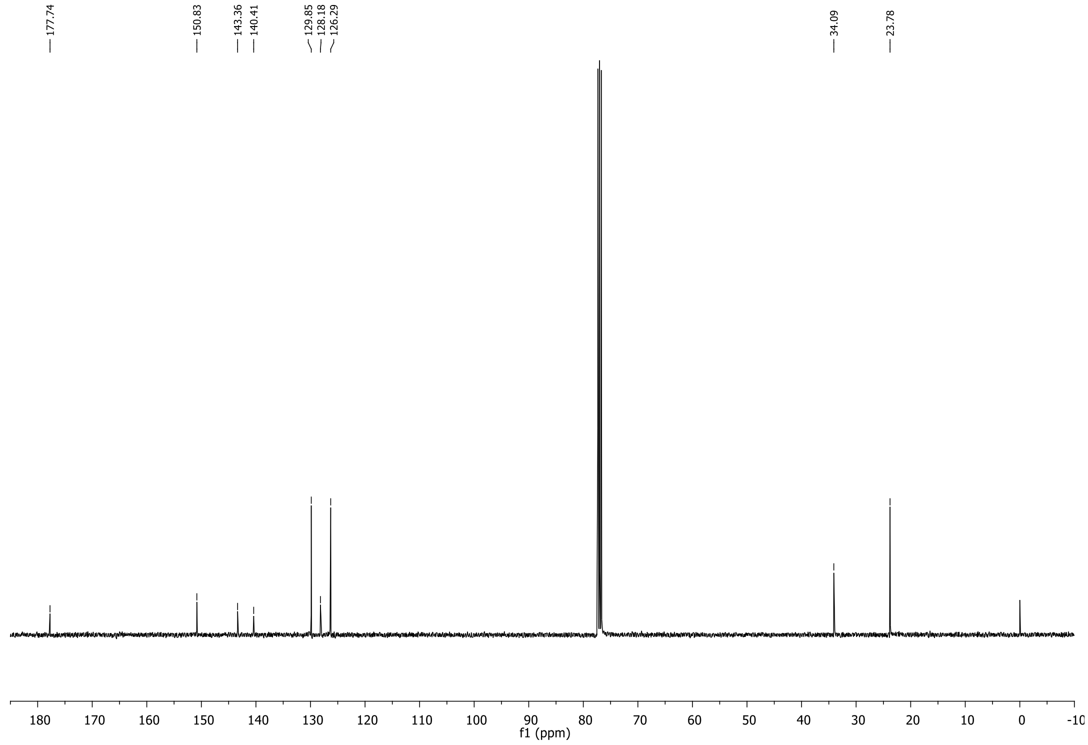

3j (^{1}H NMR, 400 MHz, $CDCl_3$)

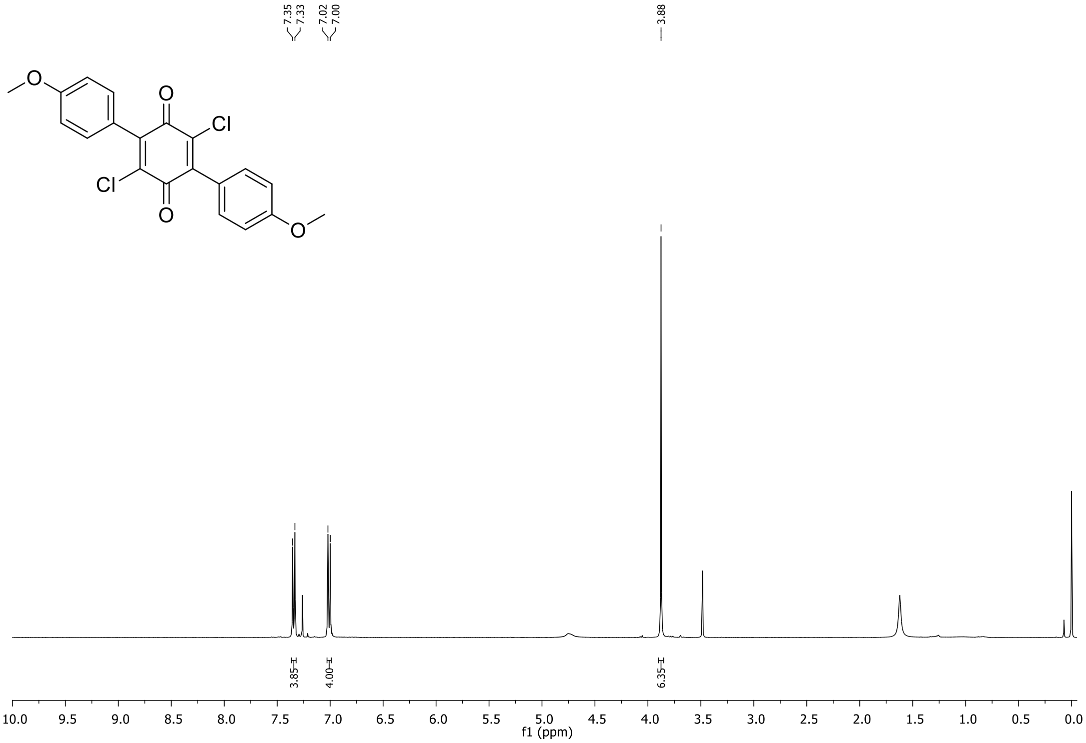

3j (^{13}C NMR, 101 MHz, $CDCl_3$)

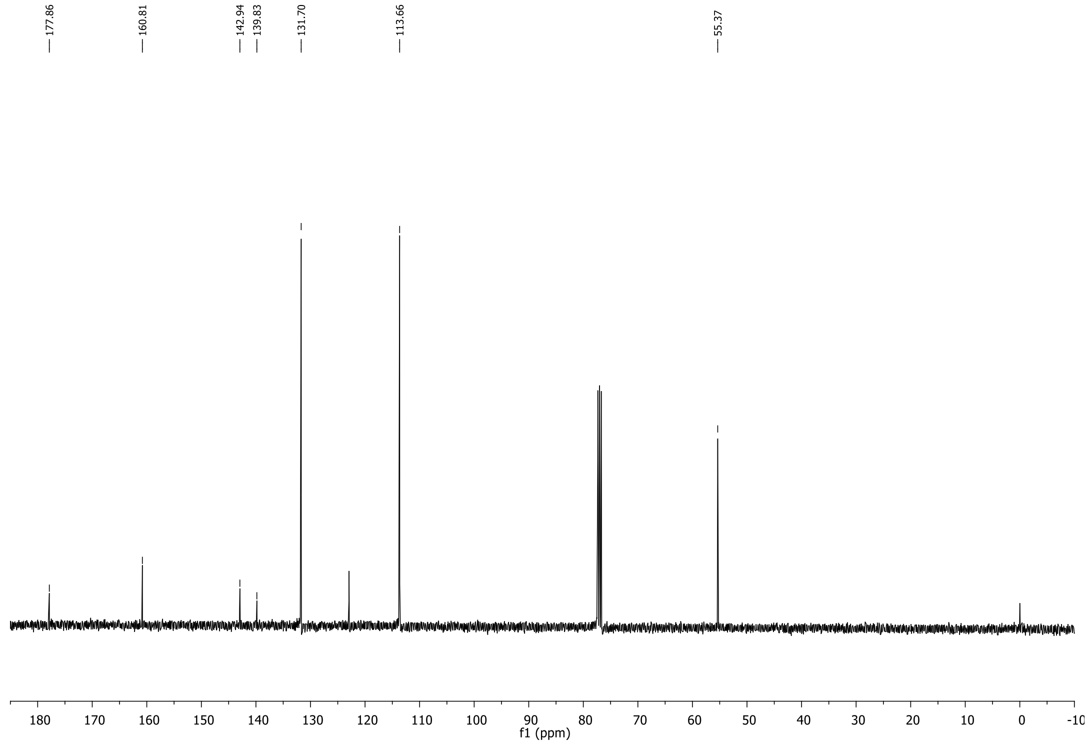

3k (^{1}H NMR, 400 MHz, DMSO)

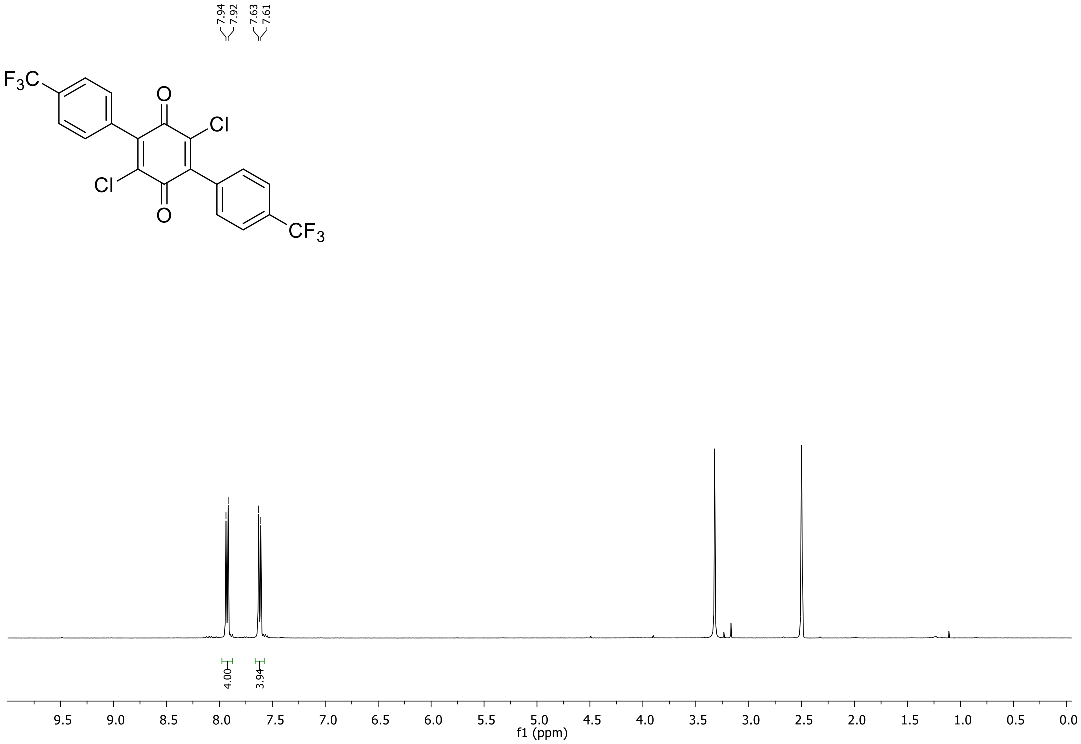

3k (^{13}C NMR, 101 MHz, DMSO)

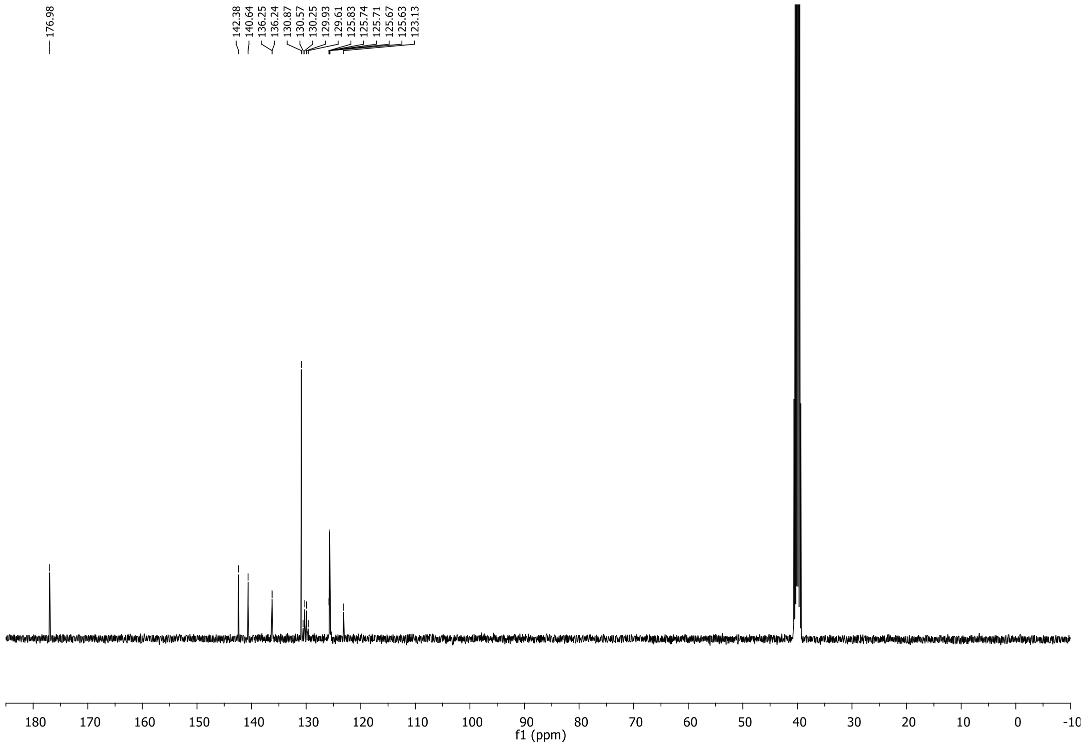

3l (^{1}H NMR, 400 MHz, $CDCl_3$)

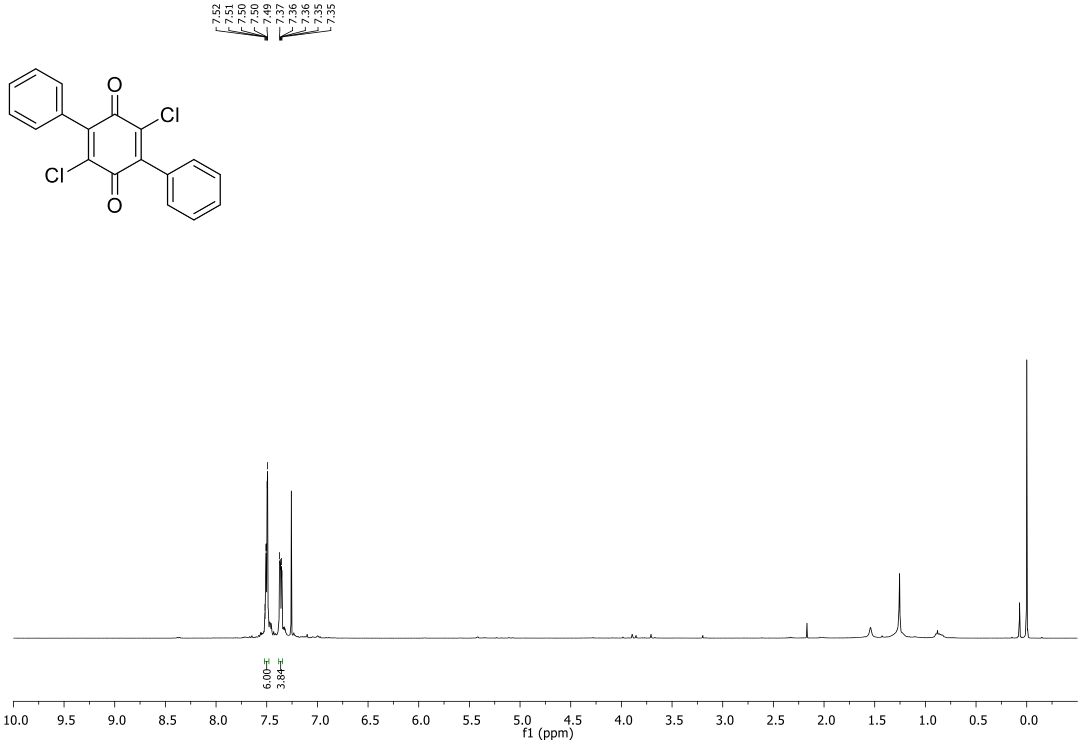

3l (^{13}C NMR, 101 MHz, $CDCl_3$)

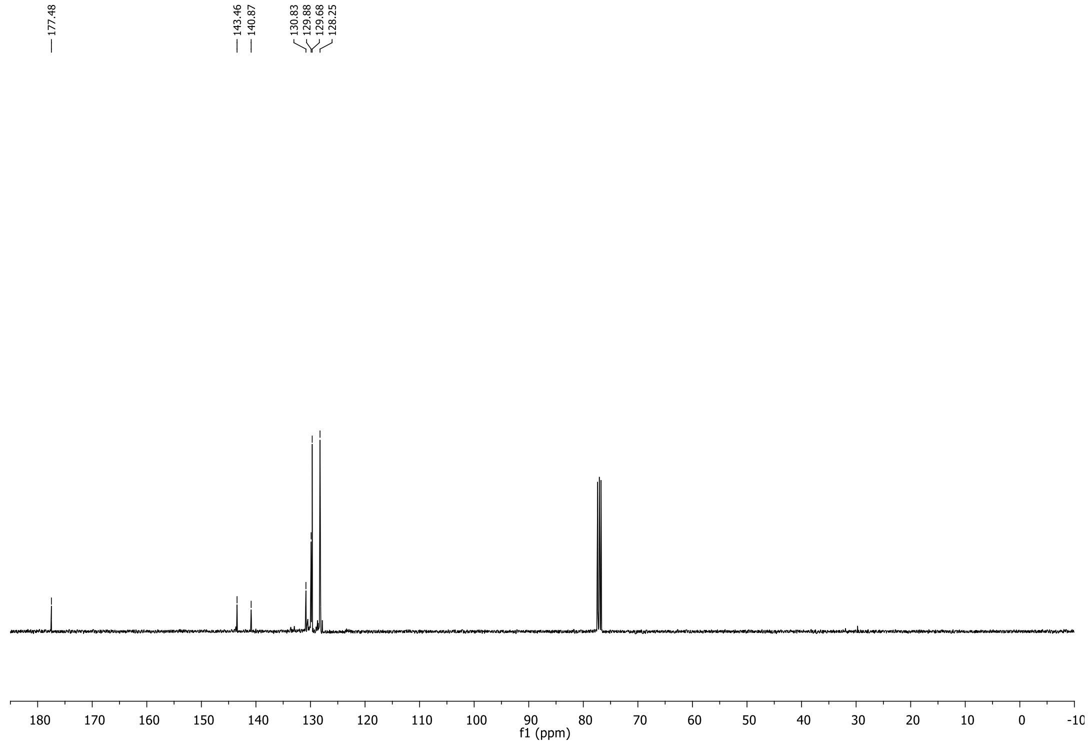

3m (^{1}H NMR, 400 MHz, DMSO)

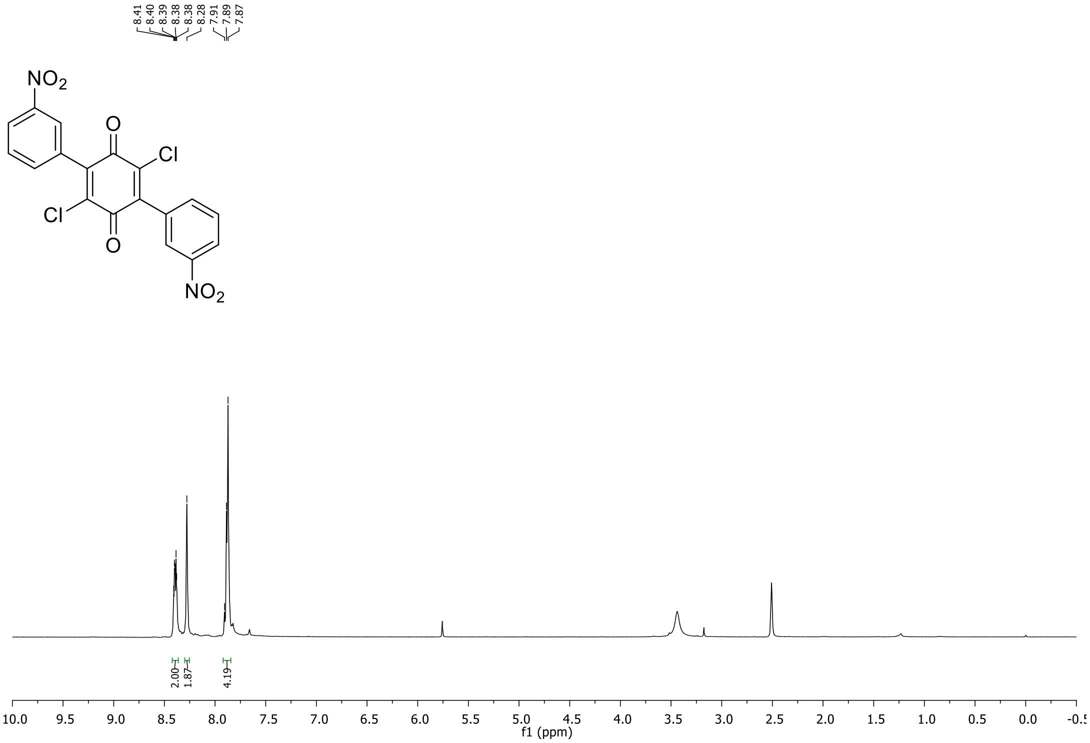

3m (^{13}C NMR, 101 MHz, DMSO)

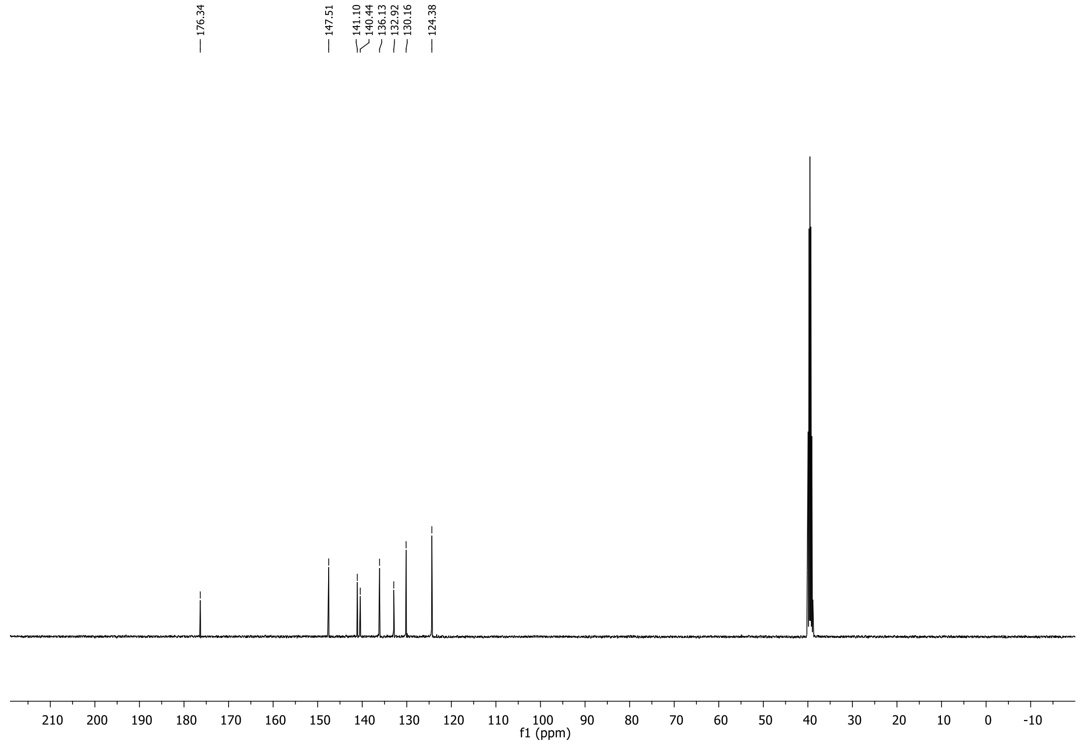

3n (^{1}H NMR, 400 MHz, DMSO)

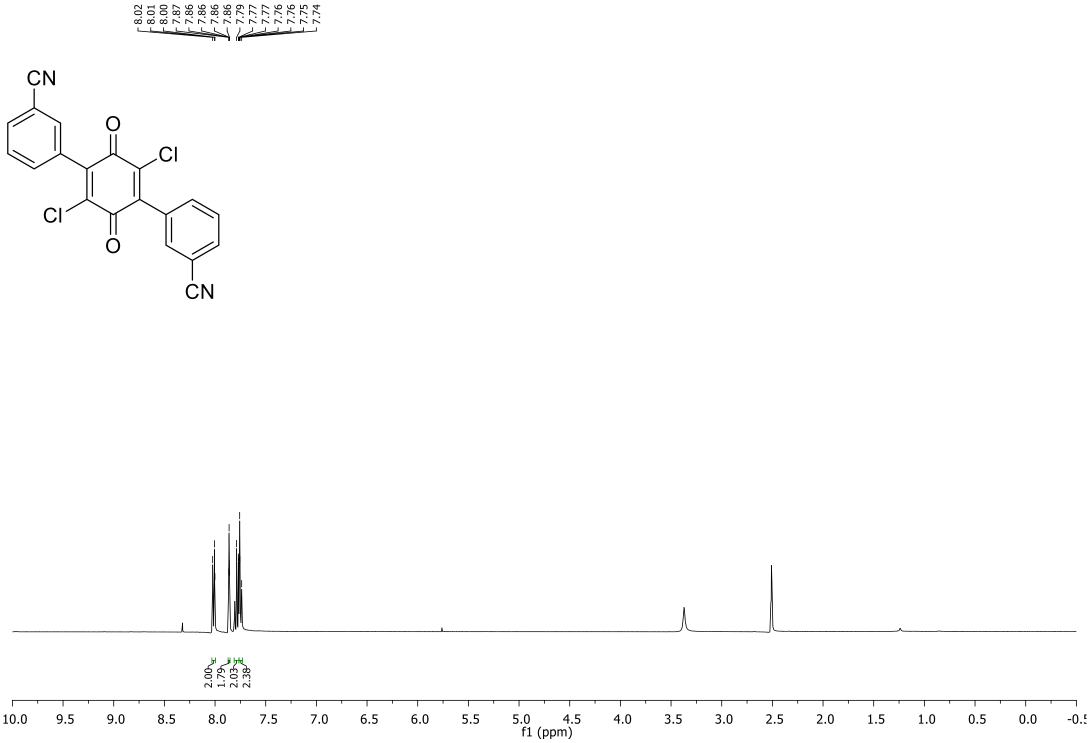

3n (^{13}C NMR, 101 MHz, DMSO)

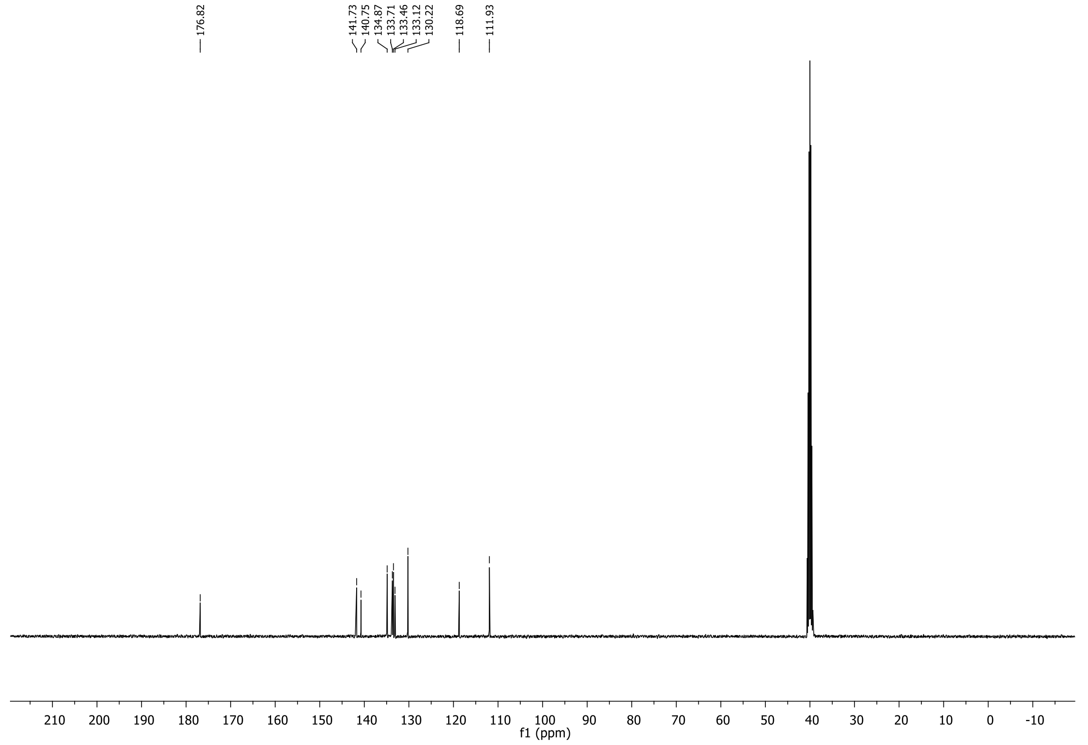

3o (^{1}H NMR, 400 MHz, $CDCl_3$)

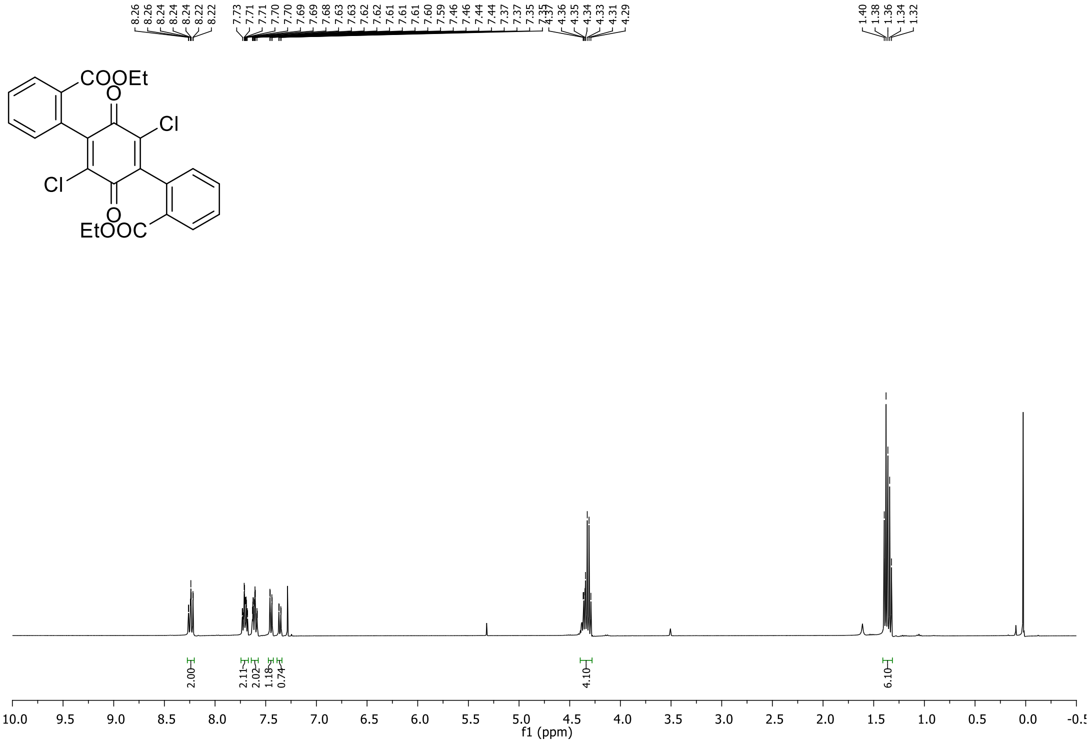

3o (^{13}C NMR, 101 MHz, $CDCl_3$)

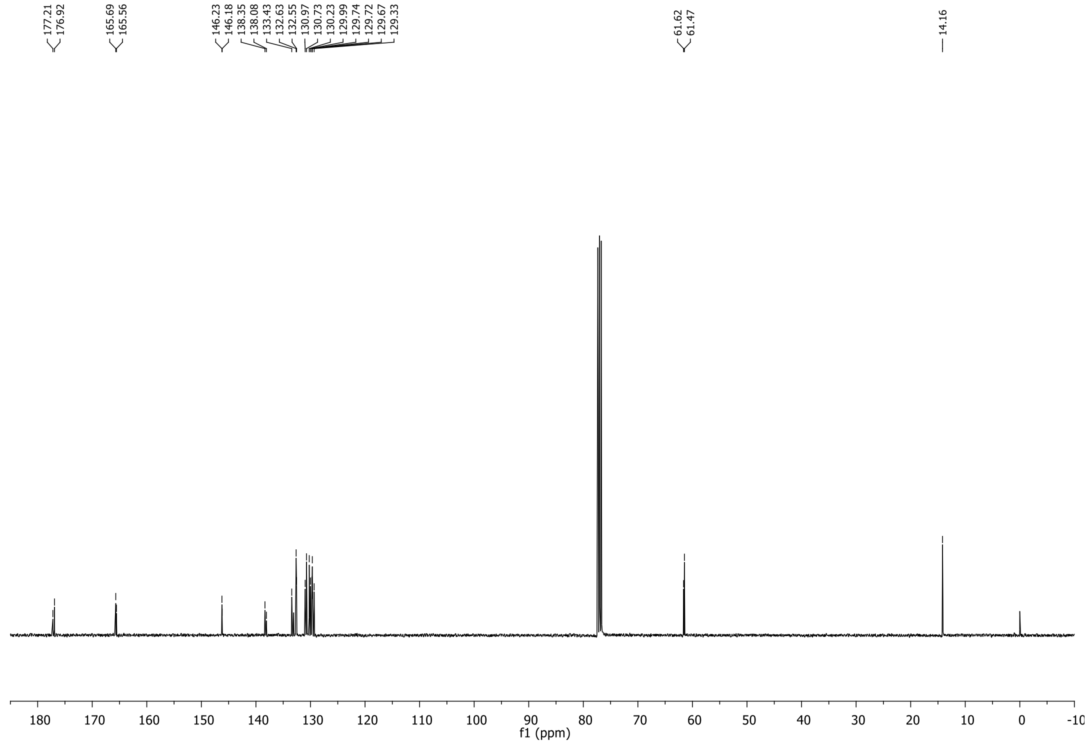

5a (^1H NMR, 400 MHz, $CDCl_3$)

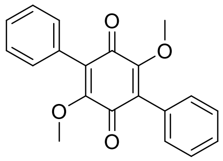

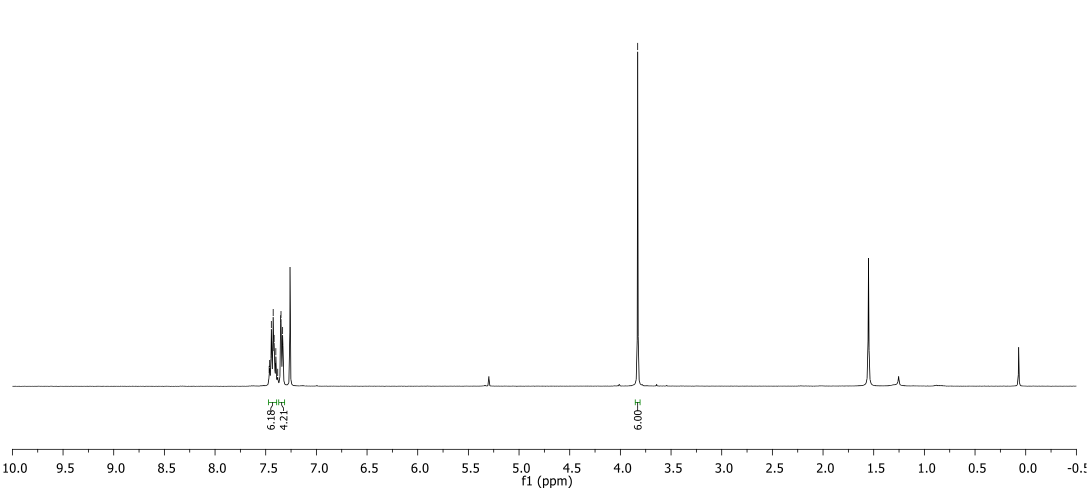

5a (^{13}C NMR, 101 MHz, $CDCl_3$)

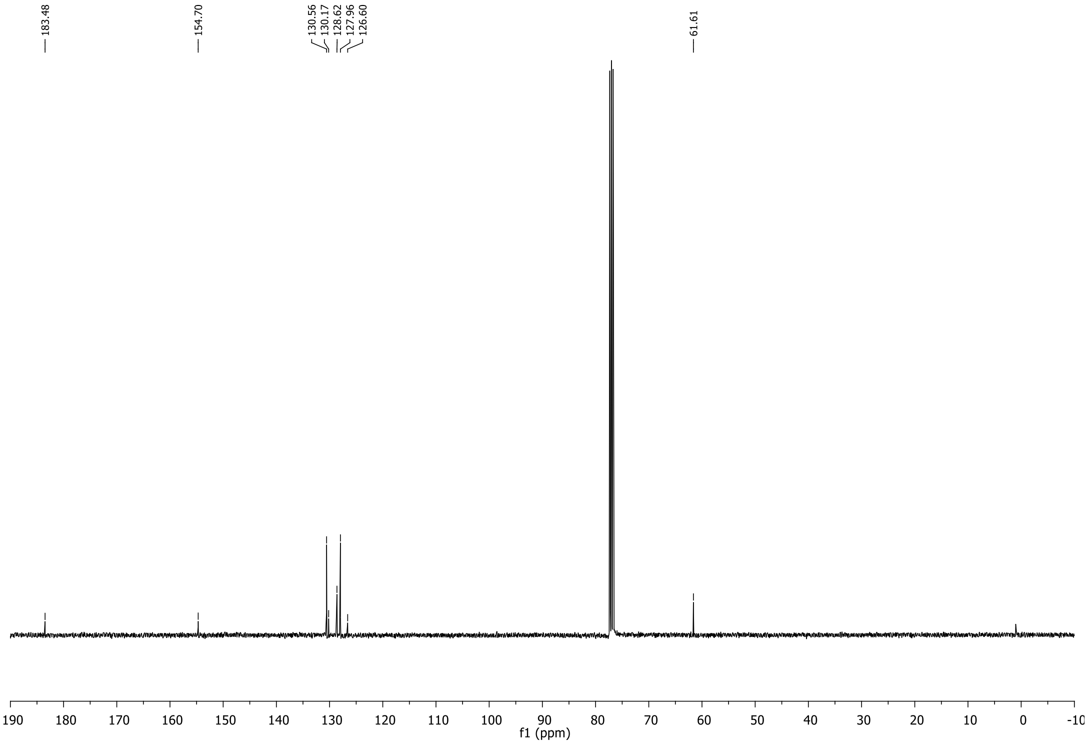

5b (^1H NMR, 400 MHz, $CDCl_3$)

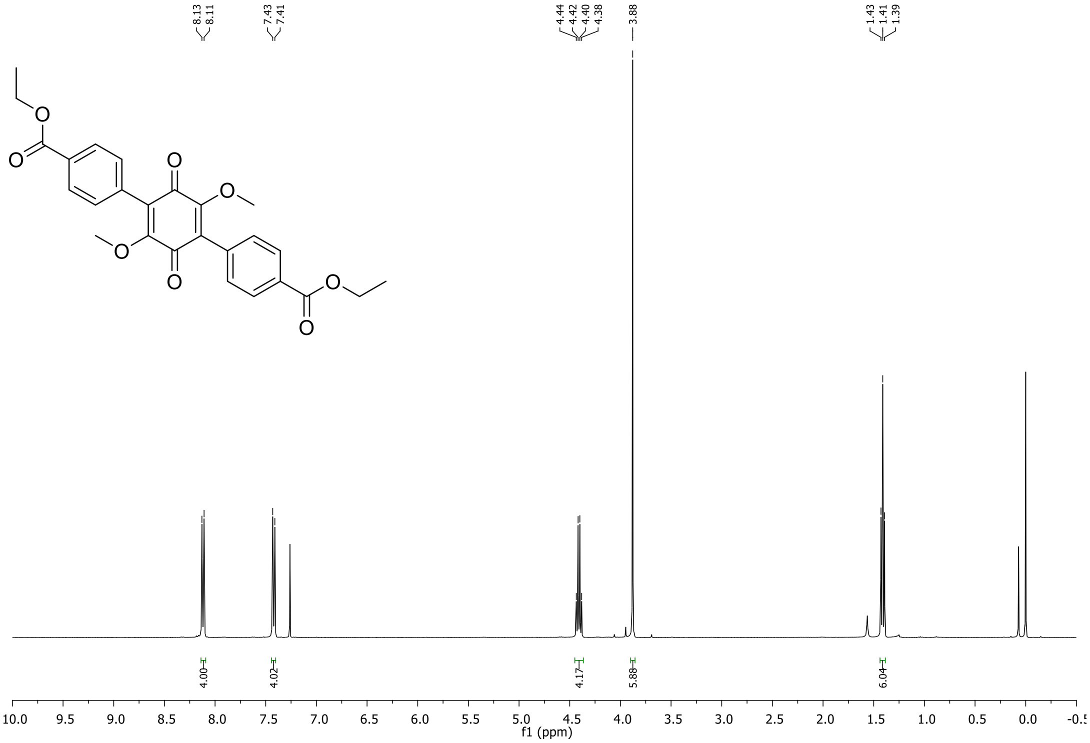

5b (^{13}C NMR, 101 MHz, $CDCl_3$)

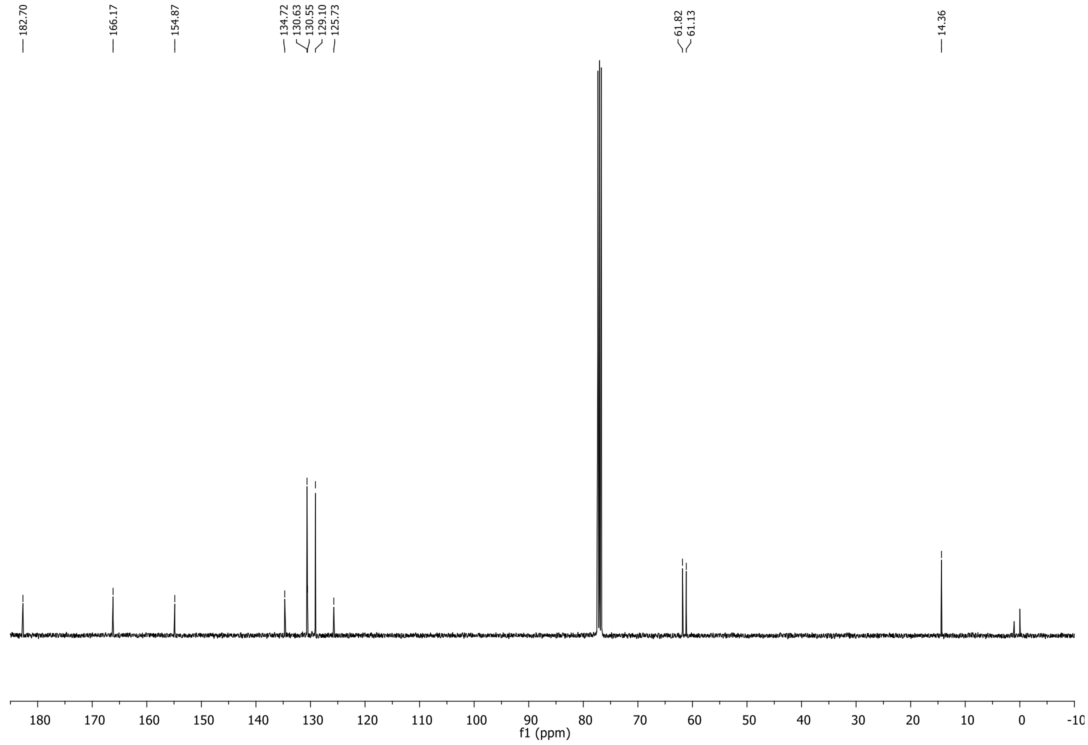

(1) Mathew, A. E.; Zee-Cheng, R. K. Y.; Cheng, C. C. *J. Med. Chem.* **1986**, *29* , 1792–1795. doi:10.1021/jm00159a041

(2) Moore, H. W.; Sing, Y. L. L.; Sidhu, R. S. *J. Org. Chem.* **1980**, *45* , 5057–5064. doi:10.1021/jo01313a009

(3) Viault, G.; Grée, D.; Das, S.; Yadav, J. S.; Grée, R. *European J. Org. Chem.* **2011**, No. 7, 1233–1241. doi:10.1002/ejoc.201001627
